# Cytoprotective and Immunomodulatory Properties of Mesenchymal Stem Cell Secretome and Its Effect on Organotypic Hippocampal Cultures in Mouse Model of Temporal Lobe Epilepsy

**DOI:** 10.3390/ijms27010265

**Published:** 2025-12-26

**Authors:** Martyna Strzelec, Jan Detka, Marta Kot, Qi Wang, Małgorzata K. Sobocińska, Jens D. Mikkelsen, Marcin Majka

**Affiliations:** 1 Department of Transplantation, Faculty of Medicine, Institute of Pediatrics, Jagiellonian University Medical College, 30-663 Krakow, Poland; martyna.strzelec@doctoral.uj.edu.pl (M.S.); jan.detka@uj.edu.pl (J.D.); marta.kot@uj.edu.pl (M.K.); mk.sobocinska@gmail.com (M.K.S.); 2 Doctoral School of Medical and Health Sciences, Jagiellonian University Medical College, 31-530 Krakow, Poland; 3 Department of Neuroscience, University of Copenhagen, 2200 Copenhagen N, Denmark; djx881@sund.ku.dk (Q.W.); jens.mikkelsen@sund.ku.dk (J.D.M.); 4 Neurobiology Research Unit, University Hospital of Copenhagen, 2100 Copenhagen Ø, Denmark

**Keywords:** mesenchymal stem cells, secretome, immunomodulatory, cell-based therapies, epilepsy, neurogenesis, inflammation, astrocytes

## Abstract

Temporal lobe epilepsy (TLE), the most common form of epilepsy, is often resistant to symptomatic treatment and characterized by persistent neuroinflammation, creating an urgent need for therapeutic strategies that can modulate early disease mechanisms. In this study, we examined the ability of the human MSC-derived secretome to influence epileptic hippocampal tissue during the latent phase of epileptogenesis using an ex vivo model. For this purpose, we characterized the MSC-derived secretome using multiplex Luminex profiling, optimized organotypic hippocampal cultures (OHCs) by evaluating cell viability, validated the pilocarpine-induced TLE model both morphologically and electrophysiologically, and investigated the influence of MSC-conditioned medium (MSC-CM) on epileptic hippocampal tissue. Using mouse-derived OHCs, we found that MSC-CM supports the preservation of nestin- and doublecortin (DCX)-positive progenitor cells, reduces NF-κB (p50/p105) levels, decreases LDH release into the culture medium, and modulates IL-6 secretion during the latent phase of epileptogenesis. Taken together, these findings suggest that the MSC-derived secretome exerts cytoprotective and context-dependent immunomodulatory effects, attenuating inflammatory signaling and cellular stress while supporting the preservation of neural progenitor markers in epileptic tissue. These properties highlight a potential phase-specific therapeutic window to modulate pathological processes during the latent phase of epileptogenesis.

## 1. Introduction

Temporal lobe epilepsy (TLE) is the most common form of epilepsy, accounting for approximately 60% of all cases [1,2]. It typically follows an initial precipitating injury—such as infection, trauma, or hypoxia—and is characterized by a seizure-free latent phase that precedes the onset of spontaneous recurrent seizures (SRS) [3]. Neuropathological features of TLE include hippocampal sclerosis, loss of inhibitory interneurons, and excitotoxic neuronal death, which collectively promote network hyperexcitability [4,5,6,7,8].

Epileptogenesis is a progressive and multifactorial pathophysiological process whereby initially non-epileptic neural networks undergo molecular, cellular, and synaptic alterations that culminate in persistent neuronal hyperexcitability and the emergence of SRS [3,9]. While epilepsy has long been considered a neuron-centric disorder, extensive evidence highlights the critical role of neuroinflammation in epileptogenesis [10]. Inflammatory responses involving activated astrocytes and microglia contribute to disease progression through disruption of the blood–brain barrier (BBB), release of pro-inflammatory cytokines, and amplification of neuronal vulnerability [11]. Neuroinflammation may be both a cause and a consequence of seizures, forming a self-perpetuating cycle that supports epileptogenic remodeling [12].

Despite advances in pharmacological interventions, approximately one-third of TLE patients remain resistant to antiepileptic drugs (AEDs) [13]. Importantly, these therapies primarily target neuronal excitability without addressing the underlying inflammatory cascade or supporting neural repair. This limitation is especially concerning in pediatric epilepsy, where long-term AED use may impair cognitive development. Therefore, alternative therapeutic strategies that combine seizure control with anti-inflammatory and neuroprotective effects are urgently needed.

Mesenchymal stem cells (MSCs), somatic multipotent cells capable of self-renewal and multilineage differentiation, have demonstrated promising therapeutic efficacy in various central nervous system (CNS) pathologies [14], including drug-resistant epilepsy [15,16]. Their beneficial effects are increasingly attributed to their secretome—a complex ensemble of soluble factors, extracellular vesicles, and regulatory molecules actively secreted into the extracellular environment, which collectively mediate immunomodulatory, neuroprotective, and tissue-repairing properties [17,18,19]. Experimental studies have demonstrated that MSCs and their derivatives exert neuroprotective and anti-inflammatory effects in epilepsy models. MSC-delivered exosomes were shown to reduce hippocampal inflammation, restore normal neurogenesis, and prevent cognitive decline and aberrant neuronal remodeling following pilocarpine-induced status epilepticus (SE) in mice [20]. Other studies have confirmed that systemically administered MSCs reduce seizure severity, promote neuroprotection, and attenuate gliosis in animal epilepsy models [21,22]. These findings highlight the therapeutic potential of MSC-based interventions in modulating epileptogenic processes. To investigate the therapeutic potential of MSC-derived factors under experimental conditions, we developed a MSC-conditioned medium (MSC-CM), which contains the full spectrum of the MSC secretome. This operational approach enabled us to evaluate the cumulative biological effects of the secreted bioactive components on epileptic tissue ex vivo. MSC-CM thus provided a controlled experimental platform for delivering and assessing the functional impact of the MSC secretome in our ex vivo model.

The pilocarpine model of TLE reproduces key pathological and clinical features of human temporal lobe epilepsy, including hippocampal hyperexcitability, a seizure-free latent period, and SRS [23,24,25]. Its extensive use in epilepsy research stems from its strong translational relevance, particularly regarding hippocampal lesions, mossy fiber sprouting, and pharmacoresistant seizure profiles [26,27,28,29,30]. In this study, a strain-adapted pilocarpine protocol was implemented to account for the heightened seizure sensitivity and vulnerability to systemic stressors characteristic of NOD-SCID mice. This adjustment aimed to improve model feasibility while preserving the core features of epileptogenesis relevant for downstream experimental analyses.

Adult neurogenesis within the hippocampus, particularly in the DG, is critically implicated in the process of epileptogenesis. Initially, the generation of new neurons may act as a reparative response to brain injury. However, in the context of epileptogenesis, this process becomes dysregulated, leading to maladaptive outcomes, mostly because newly generated cells are dislocated, which causes DG to not serve as a gatekeeper [31]. Shortly, aberrantly integrated neurons disrupt the hippocampal circuitry [32], fostering network hyperexcitability and facilitating the occurrence of SRS. This suggests that altered neurogenesis is not merely a consequence of epilepsy but can be stated as an active contributor to the establishment of the epileptic network. Additionally, epileptogenesis is characterized by two concurrent cellular processes: maladaptive increases in cell proliferation and cell apoptosis. Scientific research proves that during the latent phase, cell proliferation presents a duality. While the proliferation of progenitor cells (evidenced by increased BrdU-positive cells) remains upregulated in the DG [28], these dividing cells fail to differentiate into mature neurons, as indicated by reduced expression of NeuN [33] and microtubule-associated protein-1 (MAP-2) [4].

This study is the first to investigate the effects of human MSC-derived secretome on hippocampal tissue during the latent phase of epileptogenesis using an ex vivo approach. We characterized the MSC secretome under defined in vitro conditions, validated the pilocarpine-induced TLE model both electrophysiologically and histologically, and optimized organotypic hippocampal cultures (OHCs), which preserve the structural and cellular complexity of the hippocampus [34,35,36]. Using this platform, we examined the impact of MSC-CM on epileptic tissue from pilocarpine-treated mice (TLE model), focusing on its ability to protect neural progenitor populations, exert context-dependent immunomodulatory effects, and provide cytoprotective support during the seizure-free latent period. This underexplored phase may represent a critical therapeutic window for targeting mechanisms through which the MSC-secretome could interfere with epileptogenesis.

## 2. Results

### 2.1. Characterization of Pilocarpine-Induced Mouse Model of Temporal Lobe Epilepsy

#### 2.1.1. Electroencephalographic (EEG) Alterations in the Acute Phase of the TLE Model (Observed Within the First Hour Following Pilocarpine Administration)

**Figure 1:**
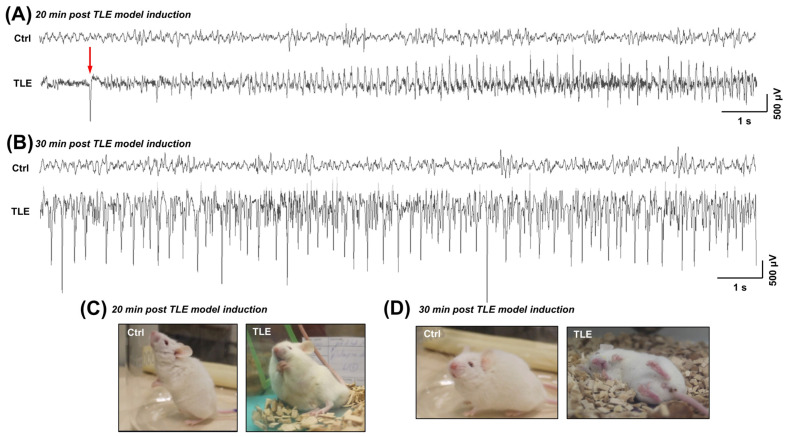
Correlation of electroencephalographic (EEG) activity and Racine’s scale behavioral manifestations in the pilocarpine-induced temporal lobe epilepsy (TLE) model. EEG signal was simultaneously acquired from a control mouse (Ctrl), which did not receive any treatment, and a TLE model mouse. Recordings were obtained at (**A**) 20 min and (**B**) 30 min after TLE model induction. Red arrow indicates pre-ictal discharge in EEG recording. (**C**,**D**) illustrate representative behavioral correlates of seizure activity corresponding to the EEG traces shown in (**A**,**B**), respectively. EEG data were sampled at 200 Hz.

To confirm that the behavioral manifestations assessed by the modified Racine scale corresponded with epileptiform EEG activity, we conducted telemetry-based EEG recordings within the first hour following pilocarpine administration. In order to verify that the epileptic behavioral phenotype correlates with neurophysiological seizure activity, EEG recordings were analyzed during the acute phase of pilocarpine-induced epileptogenesis (i.e., 20 and 30 min after intraperitoneal (i.p.) pilocarpine administration), revealing characteristic epileptiform alterations (Figure 1). Ictal spike ripples were preceded by pre-ictal discharges, as indicated by the red arrow in Figure 1A. Epileptic events were defined as EEG discharges persisting more than ten seconds, with amplitudes exceeding twice the baseline, accompanied by limbic motor symptoms corresponding to stage three or higher on the modified Racine scale. These symptoms included repeated rearing with forelimb clonus and loss of posture, generalized tonic-clonic seizures, wild running, and jumping.

#### 2.1.2. Profile of Hippocampal Parvalbumin (PV)- and Neuropeptide Y (NPY)-Positive Cells in the Latent Phase of the TLE Model (10 Days Post Model Induction)

**Figure 2:**
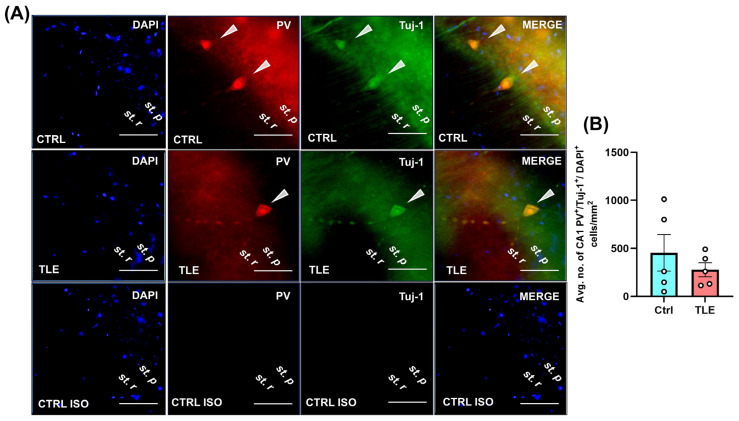
Hippocampal CA1 β-tubulin III (Tuj1) and parvalbumin (PV)—positive cells immunostaining using the Clear Lipid-exchanged Acrylamide-hybridized Rigid Imaging/Immunostaining/In situ hybridization-compatible Tissue-hYdrogel (CLARITY) method. (**A**) Representative fluorescence microscopy images of Tuj1 and PV-positive cells in control (Ctrl) and pilocarpine-induced seizures (TLE) model groups. Scale bar = 25 μm. (**B**) Quantitative analysis of PV-positive cells in the hippocampal CA1 region of Ctrl and TLE model mice. Analysis was performed using the Mann–Whitney U test. Results are presented as bar plots showing mean ± SEM; individual data points represent values from each mouse (*n* = 5 per group). Ctrl iso—isotypic control (used to assess antibody specificity), *st. p.—stratum pyramidale, st. r.—stratum radiatum.* White arrowheads indicate cell bodies with triple immunoreactivity for PV, β-tubulin III (Tuj1), and a nuclear marker.

**Figure 3:**
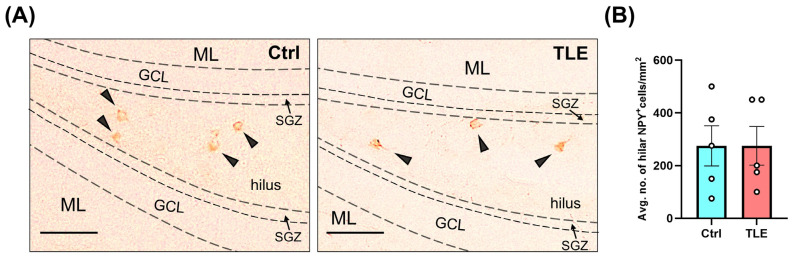
Hippocampal hilar neuropeptide Y (NPY)-positive cells visualized using immunohistochemistry. (**A**) Representative bright-field images of NPY-positive cells in the hilus of control (Ctrl) and pilocarpine-induced seizure (TLE) model mice. Scale bar = 50 μm. (**B**) Quantification of NPY-positive cells in Ctrl and TLE groups. Analysis was performed using the Mann–Whitney U test. Results are presented as bar plots showing mean ± SEM; individual data points represent values from each mouse (*n* = 5 per group). ML—molecular layer, GCL—granule cell layer, SGZ—subgranular zone. Black arrowheads indicate NPY-positive cell bodies.

To investigate the status of key inhibitory interneuron populations during the latent phase of epileptogenesis in the pilocarpine-induced TLE model, we performed immunostaining targeting PV-expressing cells in the CA1 region and NPY-positive cells in the hippocampal hilus. These interneuron subtypes were selected due to their known roles in modulating hippocampal excitability [37,38,39], and because previous reports have described changes in their profiles in experimental models of pilocarpine-induced epilepsy [20,40]. Their quantification aimed to assess potential alterations in inhibitory circuitry that may underlie or accompany early epileptogenic remodeling. For the qualitative assessment of PV-positive cells, only those exhibiting triple immunoreactivity for PV, β-tubulin III (Tuj1), and a nuclear marker were included (Figure 2, white arrows). In the evaluation of NPY-positive cells, only those localized specifically within the hippocampal hilus were considered (Figure 3).

The results demonstrated no significant differences between the control (Ctrl) and pilocarpine-treated TLE model groups in the number of PV-expressing cells in the CA1 region (Ctrl: 453.8 ± 190.6 vs. TLE: 277.6 ± 73.1; *p* = 0.7302, Mann–Whitney U test). Similarly, no significant differences were observed in the number of NPY-positive cells in the hilus (Ctrl: 274.0 ± 76.2 vs. TLE: 276.0 ± 73.3; *p* = 0.9524, Mann–Whitney U test) during the latent phase of epileptogenesis. These findings suggest that the latent phase of epileptogenesis in this strain-tailored TLE model does not involve substantial loss of PV- or NPY-expressing inhibitory interneurons in the hippocampus.

#### 2.1.3. Astrocyte Morphology Changes Under Epileptogenesis in the Latent Phase of the TLE Model (10 Days Post Model Induction)

**Figure 4:**
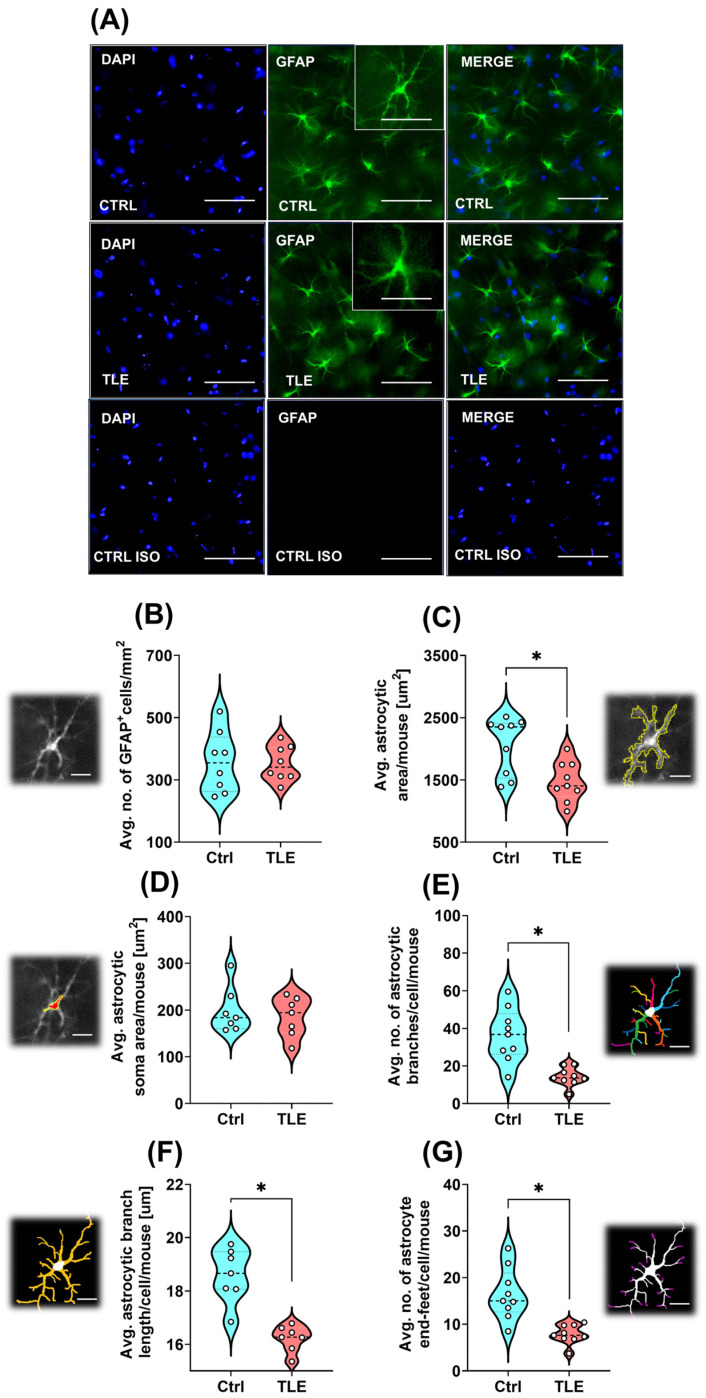
Hippocampal astrocytes in the latent phase of the mouse temporal lobe epilepsy (TLE) model visualized using the Clear Lipid-exchanged Acrylamide-hybridized Rigid Imaging/Immunostaining/In situ hybridization-compatible Tissue-hYdrogel (CLARITY) method. (**A**) Representative fluorescence images of astrocytes (green; glial fibrillary acidic protein (GFAP) immunostaining) in control (Ctrl) and pilocarpine-induced (TLE) mice. Scale bars in main panels = 25 μm; inserts = 12.5 μm. Quantitative analysis of (**B**) average number of GFAP-positive cells per mm^2^, (**C**) average astrocytic area per mouse [μm^2^], (**D**) average astrocytic soma area per mouse [μm^2^], (**E**) average number of astrocytic branches per mouse, (**F**) average astrocytic branch length per cell per mouse [μm], and (**G**) average number of astrocytic end-feet per cell per mouse. Results in violin plots are presented as mean with SEM; individual data points represent values from each mouse (*n* = 7–9). Graphic next to the bar plots represents how morphometric parameter was defined for analysis. Scale bars for graphics (**B**–**G**) = 6.25 μm. Statistical analysis was performed using the Mann–Whitney U test, where * *p* < 0.05 vs. Ctrl group. DAPI—cell nuclei, GFAP—astrocytes, Ctrl iso—isotypic control (used to assess antibody specificity).

Astrocytes were included in the analysis due to increasing evidence implicating their active involvement in the pathophysiology of epilepsy. Numerous studies have reported alterations in astrocytic morphology, reactivity, and function during epileptogenesis, including changes in branching complexity [41], organization [42], and end-feet distribution [43]. These glial cells play a critical role in modulating neuronal excitability [44], and regulating neuroinflammatory responses [45]—functions that are particularly relevant during the latent phase of epileptogenesis. Glial fibrillary acidic protein (GFAP) immunoreactivity was used to identify and visualize astrocytic populations. Morphometric analysis of astrocytes in control and pilocarpine-induced TLE model groups revealed no significant difference in the density of hippocampal astrocytes 10 days after TLE induction (Ctrl: 357.25 ± 34.58 cells/mm^2^ vs. TLE: 352.75 ± 19.95 cells/mm^2^; *p* = 0.8999, Mann–Whitney U test) (Figure 4B). However, during the latent phase of pilocarpine-induced epileptogenesis, astrocytes underwent morphological alterations, such as an overall reduction in the average size of hippocampal astrocytes (Ctrl: 2056.58 ± 151.87 μm^2^ vs. TLE: 1474.00 ± 106.54 μm^2^; *p* = 0.0142, Mann–Whitney U test) (Figure 4C), while maintaining the average size of the cell soma (Ctrl: 198.78 ± 18.56 μm^2^ vs. TLE: 185.75 ± 15.97 μm^2^; *p* = 0.9015, Mann–Whitney U test) (Figure 4D). The decrease in overall cell size is primarily attributed to a reduction in the number (Ctrl: 36.44 ± 4.70 vs. TLE: 14.28 ± 1.60; *p* = 0.0005, Mann–Whitney U test) and the length (Ctrl: 18.59 ± 0.57 μm vs. TLE: 16.22 ± 0.26 μm; *p* = 0.0006, Mann–Whitney U test) of astrocytic branches (Figure 4E and Figure 4F, respectively). Consequently, the number of astrocyte end-feet (Figure 4G) is also diminished (Ctrl: 18.30 ± 4.88 vs. TLE: 8.56 ± 0.45; *p* = 0.0003, Mann–Whitney U test).

### 2.2. Optimization of Organotypic Hippocampal Slice Culture Method

To investigate whether the viability of OHCs could be sustained until two weeks or enhanced during the first week of culture by supplementing the medium with L-glutamine and/or B-27 supplement, OHCs were established in the appropriate culture medium. Viability at selected time points (days 0, 1, 4, 7, 10, and 14) was assessed through Western blot analysis of Tuj1 protein levels—a marker of immature neurons [46] (Supplementary Figure S1A,B)—as well as colorimetric assays measuring lactate dehydrogenase (LDH) activity (Supplementary Figure S1C) and nitric oxide (NO) level (Supplementary Figure S1D) in the culture medium. The level of Tuj1 protein in OHCs showed a progressive decline, with an initial drop observed between days 0 and 1, followed by a gradual decrease over the culture period. None of the tested medium formulations significantly improved the viability of hippocampal slices. (Supplementary Figure S1A,B). These findings were corroborated by colorimetric assays, which showed no significant differences in the release of cytoplasmic LDH (Supplementary Figure S1C) or NO (Supplementary Figure S1D) into the medium by cultures obtained from healthy mice. Statistical analysis was performed using a two-way ANOVA test and Tukey’s post hoc test. Results presented as mean ± SEM, *n* = 4. Results are presented as fold change vs. the control group from each day, cultured in standard OHC medium.

### 2.3. Characterization of Mesenchymal Stem Cells Secretome

The secretion profile of MSCs used in this study was characterized by measuring the concentrations of eight selected analytes: angiopoietin-1 (ANGPT-1), brain-derived neurotrophic factor (BDNF), basic fibroblast growth factor (bFGF), bone morphogenetic protein 4 (BMP-4), glial-derived neurotrophic factor (GDNF), hepatocyte growth factor (HGF), interleukin-4 (IL-4), and neuregulin-1 β1 (NRG1 β1) using the Luminex-Multiplex method. The selected neurotrophic factors were chosen based on literature data describing their crucial roles in neuronal differentiation, dendrite and axon growth, synaptic plasticity, neuronal survival, neuroregeneration, and modulation of the neuroinflammatory response [47,48]. Presented analyte levels are shown after subtracting the values obtained from non-conditioned OHC medium to eliminate serum-related background. In the initial phase of secretome analysis, concentrations of selected analytes were determined in four MSC lines (passage five), each derived from a different human donor (Table 1). MSCs were maintained in standard OHC medium for 48 h, resulting in the generation of MSC-CM. During a 48 h culture period, all MSC lines secreted comparable amounts of the eight analytes. ANGPT-1 and HGF were detected in the highest concentrations (>1000 pg/mL). Concentrations of IL-4, NRG1 β1, BDNF, and BMP-4 remained moderately high (≥100 pg/mL), while GDNF and bFGF were present at the lowest concentrations (<100 pg/mL). However, the relative concentration ranges of all measured trophic factors were consistent across all tested donors (as displayed in Table 1).

**Figure 5:**
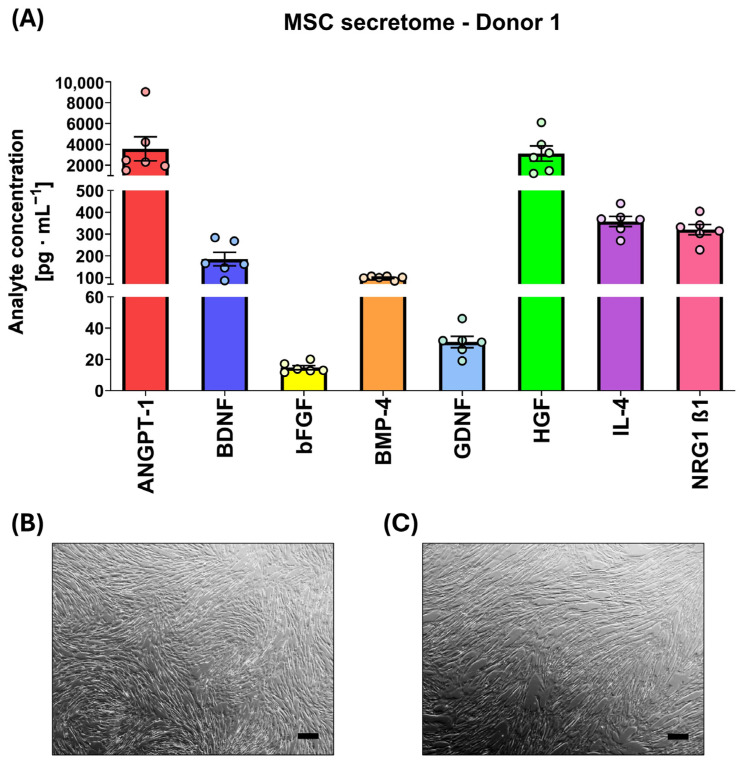
Secretion profile of MSCs isolated from Donor 1, which were selected for further ex vivo experiments involving organotypic hippocampal cultures (OHCs) (**A**). MSCs at fifth passage were cultured in standard OHC medium for 48 h. Concentrations of selected neurotrophic and anti-inflammatory factors were measured in the culture medium with multiplex immunoassay. Results are presented as mean ± SEM, number of cell cultures *n* = 6. (**B**) Representative image of MSCs isolated from Donor 1 cultured in MSC medium, before exchange to standard OHC medium, and (**C**) the same cells after 48 h of culture in standard OHC medium. Images were visualized using EVOS XL Core microscope (Life Technologies), scale bars represent 100 μm.

Since the secretion profiles were comparable across all tested lines, the MSC line derived from Donor 1 was selected for further ex vivo experiments. Analyte levels measured for Donor 1 across an extended number of culture replicates reproduced the concentration pattern reported in Table 1 (Figure 5A). MSCs remained phenotypically stable following the transition from standard MSC medium (Figure 5B) to OHC medium (Figure 5C), preserving their characteristic morphology across the entire conditioning period.

### 2.4. Assessment of Cytoprotective and Immunomodulatory Properties of the Mesenchymal Stem Cells Secretome in Organotypic Hippocampal Cultures Derived from Temporal-Lobe Epilepsy Mouse Model

#### 2.4.1. Initial Assessment of Cytoprotective and Immunomodulatory Effects of MSC-Conditioned Culture Medium on Isolated Hippocampal Tissue

Biochemical measurements of LDH, NO, and cytokine levels in OHC culturing media were conducted at days 2, 5, and 7 to align with the medium exchange schedule during the culture. Day 2 was selected as the first timepoint for analysis of culture media to assess early biochemical responses of the tissue, as reflected by the accumulation of released factors in the medium following initial exposure to the MSC-CM, and to provide sufficient incubation time for secretome–tissue interaction. OHCs were derived from pilocarpine-induced temporal lobe epilepsy (TLE) model or control (Ctrl) animals. OHCs were cultured in standard (Std) or MSC-conditioned medium (CM). The initial evaluation of the potential effect of MSC secretome on hippocampal cell viability and the immune status of the isolated brain tissue was measured by LDH activity (via tetrazolium salt assay).

**Figure 6:**
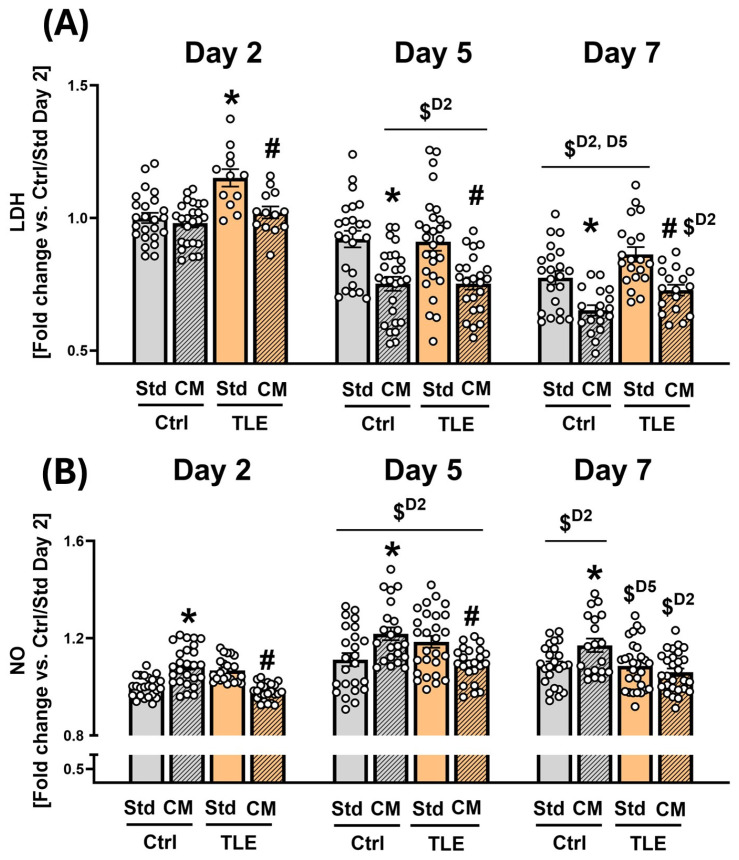
Biochemical analysis of mesenchymal stem cell (MSC) secretome influence on organotypic hippocampal cultures (OHCs) obtained from control (Ctrl) and pilocarpine-induced temporal lobe epilepsy (TLE) model group. Results of (**A**) lactate dehydrogenase (LDH) activity measured by tetrazolium salt test and (**B**) the level of nitric oxide (NO) measured by Griess test in the culture medium of OHCs isolated from Ctrl and TLE model mice cultured in standard medium (Std) and medium conditioned with mesenchymal stem cells (CM) at days 2, 5, and 7 of the culture, *n* = 13–15. All results are presented as mean ± SEM. Two-way ANOVA and Tukey’s post hoc test were used to statistically evaluate the results, where * *p* < 0.05 vs. Ctrl/Std group on the respective day, # *p* < 0.05 vs. TLE/Std group on the respective day. Differences between the same groups between individual experimental days were evaluated using one-way ANOVA and Tukey’s post hoc test, where $^D2^ *p* < 0.05 vs. same experimental group at Day 2, $^D5^ *p* < 0.05 vs. same experimental group at Day 5.

The assay revealed a statistically significant increase in LDH activity in the TLE/Std group compared to the Ctrl/Std group on the 2nd day of OHC (TLE/Std: 1.151 ± 0.025 vs. Ctrl/Std: 1.000 ± 0.019; *p* = 0.0029, Tukey’s post hoc test) (Figure 6A). In the TLE group, MSC-CM treatment significantly reduced LDH levels in the culture medium on day 2 (TLE/CM: 1.021 ± 0.030 vs. TLE/Std: 1.151 ± 0.025; *p* = 0.0392), day 5 (TLE/CM: 0.723 ± 0.038 vs. TLE/Std: 0.910 ± 0.034; *p* < 0.0001), and day 7 (TLE/CM: 0.728 ± 0.037 vs. TLE/Std: 0.863 ± 0.028; *p* = 0.0050) in comparison to the corresponding OHC group cultured in Std medium, indicating a consistent protective effect of MSC-CM over time. Similarly, in the Ctrl group, MSC-CM significantly decreased LDH levels on day 5 (Ctrl/CM: 0.752 ± 0.026 vs. Ctrl/Std: 0.920 ± 0.031; *p* < 0.0001) and day 7 (Ctrl/CM: 0.651 ± 0.020 vs. Ctrl/Std: 0.775 ± 0.026; *p* = 0.0086), but not on day 2. These findings indicate that MSC-CM exerts a time-dependent cytoprotective effect on both healthy and epileptic tissue, which corresponds with the general declining trend in LDH levels over the culture period. To further assess temporal trends in LDH release across the culture period, we conducted separate one-way ANOVA analyses within each experimental group. In the Ctrl/CM group, a significant effect of culture duration on LDH levels was observed (F(2,65) = 55.79, *p* < 0.0001). Post hoc Tukey’s test revealed a progressive and statistically significant reduction in LDH activity between Day 2 and Day 5 (*p* < 0.0001), Day 2 and Day 7 (*p* < 0.0001), as well as between Day 5 and Day 7 (*p* = 0.0078). A similar analysis in the Ctrl/Std group showed a significant effect of time (F(2,67) = 19.18, *p* < 0.0001, One-way ANOVA test), with significant reductions observed between two measured timepoints: Day 2 and Day 7 (*p* < 0.0001, post hoc Tukey’s test), and between Day 5 and Day 7 (*p* = 0.0005, post hoc Tukey’s test). In the TLE groups, LDH activity also showed a significant decline over time in both the Std medium (F(2,81) = 31.91, *p* < 0.0001, One-way ANOVA test) and CM-treated cultures (F(2,81) = 25.99, *p* = 0.0001, One-way ANOVA test). Post hoc comparisons confirmed significant decreases in the TLE/Std group between Day 2 and Day 5 (*p* < 0.0001), Day 2 and Day 7 (*p* < 0.0001), and Day 5 and Day 7 (*p* = 0.0217). In the TLE/CM group, LDH levels also significantly declined between Day 2 and Day 5 (*p* < 0.0001) and between Day 2 and Day 7 (*p* < 0.0001), but no significant difference was found between Day 5 and Day 7 (*p* = 0.8180).

The MSC secretome exerted opposing effects on NO levels in the culture medium, depending on the tissue condition. In the TLE group, CM treatment significantly reduced NO release compared to Std medium on Day 2 (TLE/CM: 0.980 ± 0.007 vs. TLE/Std: 1.068 ± 0.010; *p* = 0.0067, Tukey’s post hoc test) and Day 5 (TLE/CM: 1.094 ± 0.015 vs. TLE/Std: 1.186 ± 0.024; *p* = 0.0034, Tukey’s post hoc test), while no significant differences were observed on Day 7. This indicates that CM attenuates NO secretion in epileptic hippocampal tissue, particularly at earlier timepoints. Conversely, in the Ctrl group, CM treatment led to a significant increase in NO levels at all measured timepoints: Day 2 (Ctrl/CM: 1.087 ± 0.016 vs. Ctrl/Std: 1.000 ± 0.008; *p* = 0.0055, Tukey’s post hoc test), Day 5 (Ctrl/CM: 1.219 ± 0.026 vs. Ctrl/Std: 1.112 ± 0.027; *p* = 0.0007, Tukey’s post hoc test), and Day 7 (Ctrl/CM: 1.171 ± 0.028 vs. Ctrl/Std: 1.088 ± 0.017; *p* = 0.0191, Tukey’s post hoc test). These findings suggest a divergent, tissue-specific response to CM exposure, where MSC-derived factors promote NO release in healthy hippocampal tissue while suppressing it in epileptic tissue. One-way ANOVA revealed a significant effect of culture duration on NO levels in all experimental groups. In the Ctrl/Std group, ANOVA indicated a significant overall effect (F(2,69) = 9.526, *p* = 0.0002), and Tukey’s post hoc test showed increases between Day 2 and Day 5 (1.112 ± 0.027 vs. 1.000 ± 0.008; *p* = 0.0003) and between Day 2 and Day 7 (1.088 ± 0.017 vs. 1.000 ± 0.008; *p* = 0.0050). In the Ctrl/CM group, ANOVA showed a significant effect of time (F(2,64) = 9.177, *p* = 0.0003), with Tukey’s test indicating increases in NO levels between Day 2 and Day 5 (1.219 ± 0.026 vs. 1.087 ± 0.016; *p* = 0.0002) and between Day 2 and Day 7 (1.171 ± 0.028 vs. 1.087 ± 0.016; *p* = 0.0324). In the TLE/Std group, one-way ANOVA revealed a significant overall effect (F(2,76) = 10.910, *p* < 0.0001). Tukey’s test showed an increase in NO between Day 2 and Day 5 (1.186 ± 0.024 vs. 1.068 ± 0.001; *p* = 0.0002) and a decrease between Day 5 and Day 7 (1.087 ± 0.019 vs. 1.186 ± 0.024; *p* = 0.0010). In the TLE/CM group, ANOVA confirmed a significant time effect (F(2,71) = 18.08, *p* < 0.0001). Tukey’s test revealed significant increases in NO levels between Day 2 and Day 5 (1.094 ± 0.015 vs. 0.978 ± 0.007; *p* < 0.0001) and between Day 2 and Day 7 (1.060 ± 0.015 vs. 0.980 ± 0.007; *p* = 0.0002).

#### 2.4.2. Investigation into the Protective Effects of Mesenchymal Stem Cell Secretome on Neural Progenitor Markers in Isolated Hippocampal Slices

In contrast to culturing medium analysis, protein analysis by Western blot was performed on tissue lysates collected at days 1, 4, and 7, reflecting the start, mid-point, and endpoint of the culture period. These distinct timepoints were selected to capture early and cumulative cellular responses to the MSC-CM within the tissue itself, independently of the media exchange cycle. The MSC secretome utilized during in vitro studies was shown to contain a considerable concentration of neurotrophic factors (Figure 5A). Therefore, to verify whether the MSC secretome exerts a protective effect on hippocampal progenitor cells, Western blot analysis was performed to determine the levels of nestin—a protein marker of neural stem cells and early progenitor cells, doublecortin (DCX)—a marker of immature migrating neurons, and Tuj1, which is expressed in newly differentiated and mature neurons. These proteins were quantified in OHC homogenates derived from the TLE mouse model and cultured in Std and CM for 1, 4, and 7 days.

**Figure 7:**
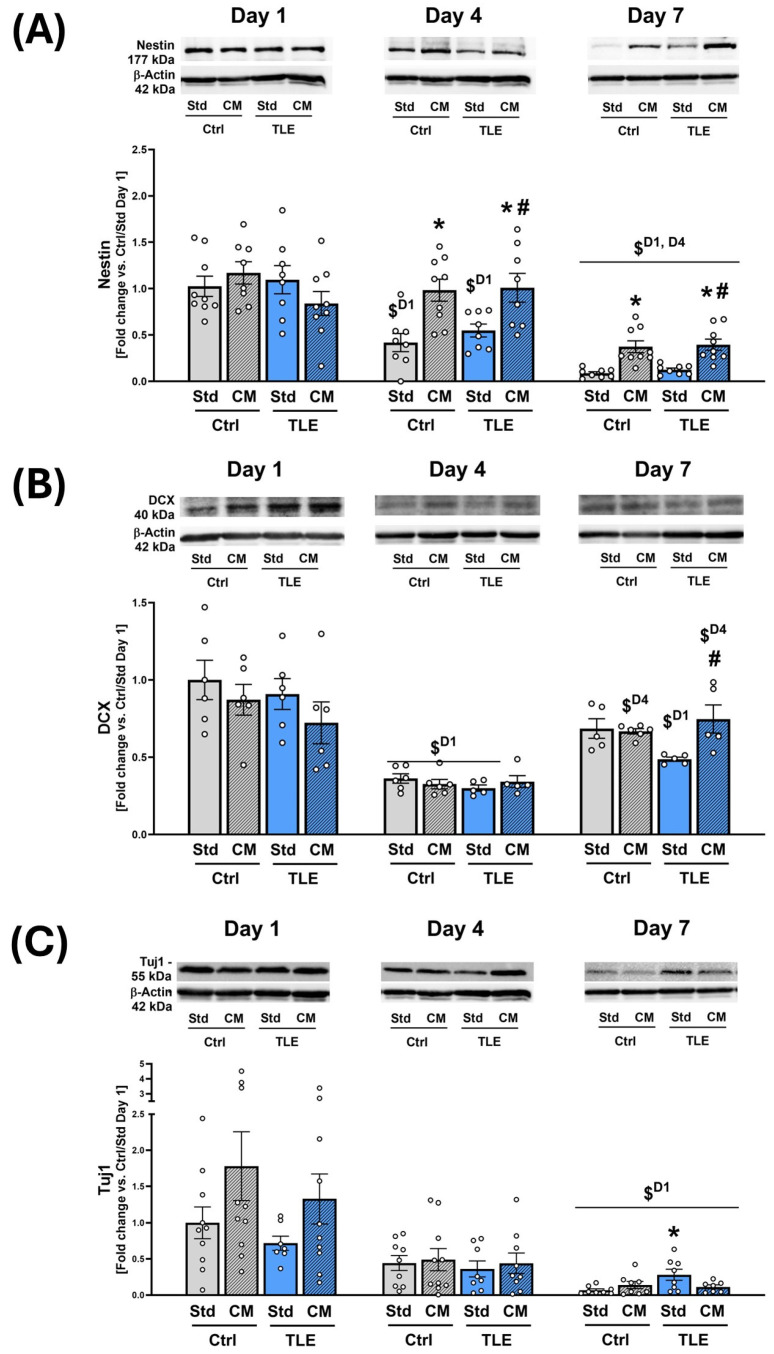
Results of Western blot analysis in organotypic hippocampal cultures (OHCs) homogenates showing the amounts of selected cellular markers, characteristic of different stages of neuronal cell differentiation. Levels of nestin (**A**), doublecortin (DCX) (**B**) and β-tubulin III (Tuj1) (**C**) in pilocarpine-induced temporal lobe epilepsy (TLE) model group and control (Ctrl) animals, after 1, 4 and 7 days of culture in standard medium (Std) and medium conditioned with mesenchymal stem cells (CM), *n* = 8–9 per group for nestin; *n* = 5–6 per group for DCX; *n* = 7–10 per group for Tuj1. All results are presented as means ± SEM. Two-way ANOVA and Tukey’s post hoc test were used to statistically evaluate the results, where * *p* < 0.05 vs. Ctrl/Std group on the respective day, # *p* < 0.05 vs. TLE/Std group on the respective day. Differences between the same groups between individual experimental days were evaluated using one-way ANOVA and Tukey’s post hoc test, where $^D1^ *p* < 0.05 vs. same experimental group at Day 1, $^D4^ *p* < 0.05 vs. same experimental group at Day 4.

Two-way analysis of variance (ANOVA) showed no significant effect of TLE or CM on nestin levels on the first day of the experiment (TLE: F(1,30) = 0.7648 *p* = 0.388771; CM: F(1,30) = 0.0023 *p* = 0.9620). On Day 4, however, its content in OHCs in the Ctrl/Std and TLE/Std groups decreased significantly, when compared to Day 1 (Ctrl/Std: 0.496 ± 0.223 vs. 1.000 ± 0.327 at Day 1 *p* = 0.0006; TLE/Std: 0.548 ± 0.198 vs. 1.058 ± 0.419 at Day 1 *p* = 0.0038). In the case of groups maintained in conditioned medium (CM), there was no decrease in nestin levels on Day 4 compared to Day 1 (Ctrl/CM Day 4: 0.983 ± 0.356 vs. 1.170 ± 0.340 at Day 1 *p* = 0.308011; TLE/CM Day 4: 1.009 ± 0.439 vs. 0.924 ± 0.312 at Day 1 *p* = 0.8599). Moreover, nestin content on fourth day of experiment was significantly higher in both groups maintained in CM compared to OHCs cultured in standard (Std) medium (*p* = 0.0244 for Ctrl/CM at Day 4 vs. Ctrl/Std; *p* = 0.0359 for TLE/CM vs. TLE/Std at Day4 and *p* = 0.0166 vs. Ctrl/Std at Day 4) (Figure 7A).

On the seventh day of culture, the level of nestin in all groups decreased significantly compared to the previous days of the experiment (Ctrl/Std: 0.088 ± 0.047 vs. 0.496 ± 0.223 at Day 4 *p* = 0.0061 and vs. 1.000 ± 0.327 at Day 1 *p* = 0.0001; Ctrl/CM: 0.373 ± 0.190 vs. 0.983 ± 0.356 Day 4 *p* = 0.0007 and vs. 1.170 ± 0.340 at Day 1 *p* = 0.0002; TLE/Std: 0.126 ± 0.050 vs. 0.548 ± 0.198 at Day 4 *p* = 0.0160 and vs. 1.058 ± 0.419 at Day 1 *p* = 0.0001; TLE/CM: 0.395 ± 0.180 vs. CM Day 4: 1.009 ± 0.439 *p* = 0.0028 and vs. 0.924 ± 0.312 at Day 1 *p* = 0.0093). However, similarly to the fourth day of culture, nestin content in OHCs maintained in conditioned medium was significantly higher compared to hippocampal slices maintained in standard medium (Std) (*p* = 0.0016 for Ctrl/CM at Day 7 vs. Ctrl/Std; *p* = 0.0030 for TLE/CM vs. TLE/Std at Day 7 and *p* = 0.0008 vs. Ctrl/Std at Day 7). These data indicate that nestin levels remained significantly elevated in CM-treated slices in the late culture phase, in both TLE and control groups, relative to Std conditions (Figure 7A).

Twenty-four hours after establishment of OHC cultures (at Day 1), the levels of DCX also remained unchanged under the influence of both TLE and CM (F(1,20) = 1.0599 *p* = 0.3155 for TLE; F(1,20) = 1.8234 *p* = 0.1920 for CM). At Day 4, significant decreases of DCX levels were observed in Ctrl/Std, Ctrl/CM and TLE/Std groups in comparison with corresponding groups at Day 1 (Ctrl/Std: 0.3622 ± 0.0740 vs. 1.000 ± 0.3120 at Day1 *p* = 0.0004 Ctrl/CM: 0.3259 ± 0.0742 vs. 0.8715 ± 0.2435 at Day 1 *p* = 0.0002; TLE/Std: 0.3004 ± 0.04514 vs. 0.9089 ± 0.2444 at Day 1 *p* = 0.0003). However, no significant decrease was observed for the TLE/CM group (0.3426 ± 0.0843 vs. 0.7226 ± 0.3312 at Day1 *p* = 0.0628). On the fourth day of culture, two-way ANOVA did not reveal significant main effects of any of the studied factors (TLE: F(1,18) = 0.5479 *p* = 0.46871; CM: F(1,18) = 0.009 *p* = 0.9253). On the seventh day of culture, however, the analysis of variance showed a significant effect of CM (F(1,17) = 5.0264 *p* = 0.0386) and TLE*CM interaction (F(1,17) = 6,6657 *p* = 0.019396) on the level of DCX in isolated hippocampal tissue. In hippocampal slices isolated from mice with TLE, cultured in MSC-CM (TLE/CM), the level of DCX was significantly higher, when compared to OHCs from the same group of mice maintained in standard medium (TLE/Std) (TLE/CM: 0.7470 ± 0.2236 vs. 0.4870 ± 0.0299 for TLE/Std *p* = 0.0185) The amount of DCX at seventh day of OHC culture was also significantly increased in TLE/CM group, when compared to fourth experimental day (TLE/CM: 0.7470 ± 0.2236 vs. 0.3426 ± 0.0843 at Day 4 *p* = 0.0470) (Figure 7B).

One-way ANOVA and Tukey post hoc tests revealed significant decrease of Tuj1 levels in all experimental groups at Day 7 (Ctrl/Std: 0.068 ± 0.053 vs. 1.000 ± 0.698 *p* = 0.0033 at Day 1; Ctrl/CM: 0.139 ± 0.140 vs. 1.780 ± 1.505 *p* = 0.0027; TLE/Std: 0.282 ± 0.217 vs. 0.718 ± 0.260 *p* = 0.0162; TLE/CM: 0.114 ± 0.073 vs. 1.328 ± 1.087 *p* = 0.0126). Two-way analysis of variance of Tuj1 Western blot results did not reveal any significant main effects of TLE and CM at first and fourth days of OHC culture (TLE: F(1,33) = 1.1200 *p* = 0.2976; CM: F(1,33) = 4.0170 *p* = 0.0533 at Day 1; TLE: F(1,32) = 0.6216p = 0.2485; CM: F(1,32) = 0.2246 *p* = 0.6388 at Day 4). On the seventh day, however, an interaction of TLE*CM was observed (F(1,27) = 6.06370 *p* = 0.0205) and a significant increase in TLE/Std group in comparison with OHC isolated from control mice cultured in standard medium (Ctrl/Std) (*p* = 0.0277) (Figure 7C).

#### 2.4.3. Evaluation of Immunomodulatory Action of Mesenchymal Stem Cell Secretome in Organotypic Hippocampal Cultures

**Figure 8:**
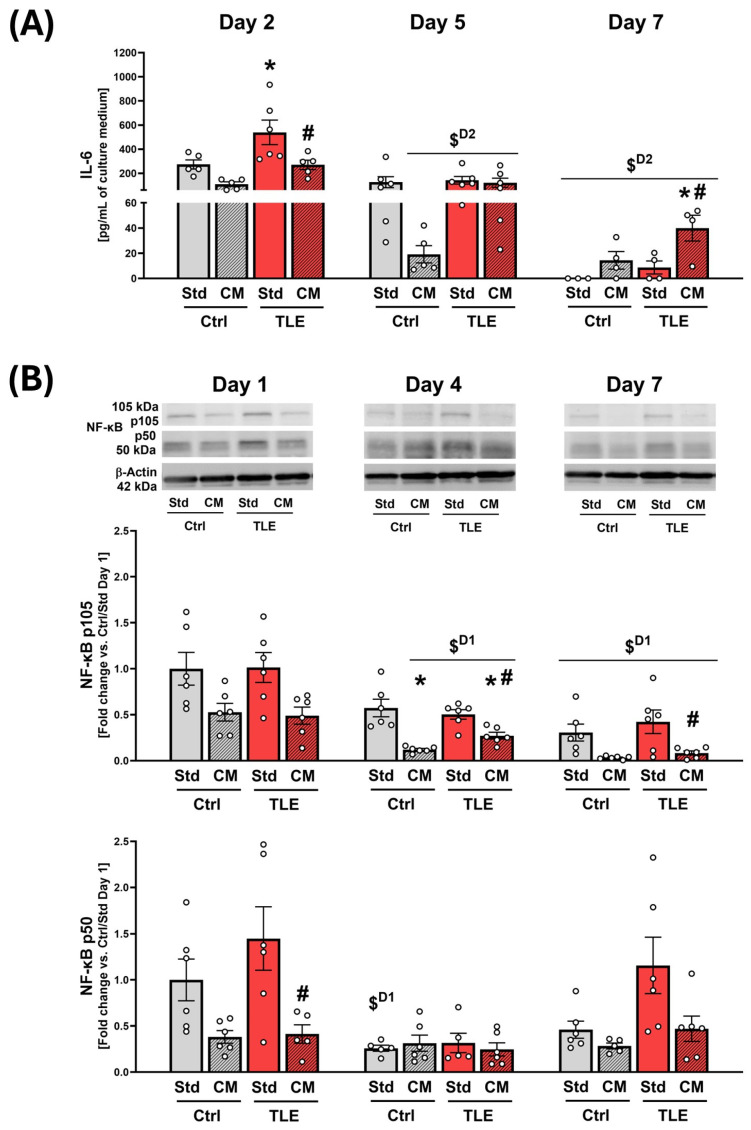
Biochemical and molecular analysis of immunomodulatory and inflammatory markers in organotypic hippocampal cultures (OHCs). (**A**) Level of interleukin 6 (IL-6) in the OHC medium in Std and CM measured using a multiplex immunoassay. Results are presented as pg/mL, *n* = 3–6/group. (**B**) Level of precursor (p105) and active form (p50) of nuclear factor kappa B (NF-κB), determined by Western blot analysis; *n* = 5–6 per group. All results are presented as means ± SEM. Two-way ANOVA and Tukey’s post hoc test were used to statistically evaluate the results, where * *p* < 0.05 vs. Ctrl/Std group on the respective day, # *p* < 0.05 vs. TLE/Std group on the respective day. Differences between the same groups between individual experimental days were evaluated using one-way ANOVA and Tukey’s post hoc test, where $^D1^ *p* < 0.05 vs. same experimental group at Day 1, $^D2^ *p* < 0.05 vs. same experimental group at Day 2.

Multiplex luminometric immunoassay was performed to determine an array of pr-inflammatory and anti-inflammatory cytokines secreted by OHCs during the culture course. Among the cytokines tested, only IL-6 was present at concentrations sufficient to be detected in the culture medium by the applied assay method. IL-6 secretion from hippocampal tissue showed divergent responses to MSC secretome treatment, with contrasting trends observed between the initial and final culture periods. On the second day of culture, IL-6 concentration was significantly elevated in OHCs isolated from the TLE model group, when compared with the respective control group (Ctrl/Std) (TLE/Std: 539.345 ± 248.563 pg/mL vs. 272.846 ± 84.305 pg/mL for Ctrl/Std; *p* = 0.0396), and this increase was reversed in hippocampal slices cultured in CM (TLE/CM: 269.692 ± 87.644 pg/mL vs. 539.345 ± 248.563 pg/mL for TLE/Std *p* = 0.0369) (Figure 8A). Over the course of 7 days, secretion of IL-6 decreased significantly compared to day 1 (Ctrl/Std: 0.000 ± 0.000 pg/mL vs. 272.846 ± 84.305 pg/mL at Day 2 *p* = 0.0096; Ctrl/CM: 14.351 ± 13.943 vs. 109.806 ± 42.249 pg/mL at Day 2 *p* = 0.0016; TLE/Std: 8.713 ± 10.331 pg/mL vs. 539.345 ± 248.563 pg/mL at Day 2 *p* = 0.0014; TLE/CM: 39.935 ± 20.455 pg/mL vs. 269.692 ± 87.644 pg/mL at Day 2 *p* = 0.0040). On day 7, the release of IL-6 from OHCs remained at the highest level in slices isolated from TLE model mice cultured in CM, which was significantly increased in comparison with the corresponding group cultured in Std medium (TLE/CM: 39.935 ± 20.455 vs. 8.713 ± 10.331 for TLE/Std *p* = 0.0397) (Figure 8A).

For further verification of the anti-inflammatory properties of MSC secretome, the levels of both the active form of nuclear factor kappa-light-chain-enhancer of activated B cells (NF-κB) p50 and its precursor protein p105 were also determined in OHC tissue homogenates. NF-κB is a transcription factor indicative of an ongoing inflammatory process. It is activated in response to excitotoxicity, as well as oxidative or metabolic stress, and is also known to be upregulated in the epileptic brain [49].

In the case of NF-κB p105, the precursor form of NF-κB, analysis of variance showed a significant effect of CM in OHCs on the 4th and 7th day of culture (F(1,20) = 35.0869 *p* = 0.000009 for Day 4; F(1,20) = 14.7703 *p* = 0.0010 for Day 7). After four days, the level of p105 was significantly decreased in CM-treated OHCs isolated from both Ctrl and TLE model animals, in comparison with corresponding control groups cultured in Std medium (Ctrl/CM: 0.121 ± 0.032 vs. 0.574 ± 0.234 for Ctrl/Std at Day 4 *p* = 0.0003; TLE/CM: 0.273 ± 0.090 vs. 0.504 ± 0.127 for TLE/Std at Day 4 *p* = 0.0469 and *p* = 0.0074 vs. Ctrl/Std at Day 1). On day 7, however, this effect was restricted only to OHCs from the TLE model group (TLE/CM: 0.085 ± 0.055 vs. 0.424 ± 0.314 for TLE/CM at Day 7 *p* = 0.0322) (Figure 8B).

The level of the active form of the transcription factor NF-κB (p50) in OHCs cultured in CM was lower compared to those cultured in Std medium on each day of culture in OHCs from both the Ctrl group and the TLE model group. However, the analysis of variance showed a significant effect of CM only on the first day of culture (F(1,19) = 13.6981 *p* = 0.001516). A post hoc test revealed a statistically significant decrease in the level of NF-κB p50 under the influence of MSC secretome-enriched medium in OHCs isolated from animals treated with pilocarpine, compared to the corresponding group cultured in the standard medium (TLE/CM: 0.414 ± 0.223 vs. 1.448 ± 0.839 for TLE/Std *p* = 0.0298) (Figure 8B).

## 3. Discussion

In this study, we employed OHCs derived from a well-established mouse model of TLE, implemented using a modified pilocarpine protocol tailored to the strain-specific sensitivity of NOD-SCID mice. The 10-day timepoint post-pilocarpine administration was selected as a well-established period within the latent phase of epileptogenesis, preceding the onset of SRS, yet marked by active pathological remodeling. At this stage, molecular and cellular changes—such as neuroinflammation [50], glial activation [51], cell loss [52] and early synaptic reorganization [43]—are initiated and have been documented in both human tissue and rodent models of TLE. These early events are believed to shape the subsequent transition into the chronic epileptic state and thus represent a critical therapeutic window. By targeting this phase, we aimed to investigate whether MSC-derived secretome can interfere with pathogenic cascades underlying epileptogenesis. Our findings demonstrate that MSC-derived secretome exerts cytoprotective effects and supports the preservation of neural cells, alongside context-specific immunomodulatory effects in hippocampal slice cultures. This is evidenced by the prevention of the decline in neural progenitor markers, reduced NF-κB (p50/p105) levels, and a potential immunomodulatory influence on IL-6 secretion. Decreased LDH release further supports its role in reducing cellular stress. Epileptic phenotype, elicited using a strain-specific tailored TLE protocol, was confirmed by EEG and behavioral seizures during the acute phase. No significant changes in CA1 parvalbumin-positive or hilar NPY-positive neurons were observed at day 10, indicating preservation of these interneuron subtypes under the specific conditions of the applied protocol. Culture optimization revealed that L-glutamine and/or B-27 supplementation did not improve neuronal viability in adult OHCs, and morphometric analysis showed astrocytic features of senescence, which may compromise their function during epileptogenesis.

### 3.1. Characterization of Pilocarpine-Induced Temporal Lobe Epilepsy Mouse Model and Organotypic Hippocampal Slice Cultures

Our research was conducted using a well-established mouse model of TLE induced by i.p. administration of pilocarpine [23], employing a strain-tailored protocol. Radiotelemetric EEG analysis confirmed the presence of epileptiform activity, supporting the occurrence of neurophysiological alterations in animals exhibiting behavioral seizures. To characterize the profile of selected hippocampal inhibitory cell populations during the latent phase of epileptogenesis, we quantified parvalbumin (PV)-positive cells in the CA1 region and neuropeptide Y (NPY)-positive interneurons in the hilus using immunofluorescence and immunohistochemistry. The latent phase of epileptogenesis is commonly associated with a progressive loss of PV-positive interneurons [53,54,55] and reductions in NPY-positive cells, observed in both the pilocarpine model [56] and other TLE models [57], potentially contributing to increased network excitability. These findings differ from our results, as we did not observe any significant changes in the number of PV^+^ cells in CA1 or NPY^+^ cells in the hilus during the latent phase. This discrepancy may be attributed to our specific TLE induction protocol, in which the initial seizure was promptly terminated with diazepam, limiting its duration and preventing excessive excitotoxicity. The reduced seizure burden may have preserved these interneuron populations—particularly PV^+^ cells, which are known for their relative resistance to excitotoxic damage [53]. Consequently, the potential effects of the MSC secretome on these cell populations were not further investigated.

Following the first seizure onset, enhanced cell proliferation within the dentate gyrus (DG) contributes not only to neurogenesis but also to increased astrogenesis, potentially promoting reactive gliosis [58]. Given the role of astrocytic activation in epileptogenic remodeling, we further examined structural alterations in these glial cells during the latent phase of epileptogenesis by performing morphometric analysis of GFAP-positive cells using fluorescence microscopy. Our analysis revealed that astrocytes undergo structural changes consistent with cellular senescence. Specifically, a reduction in the total number and length of astrocytic branches, as well as a decreased number of end-feet formations, was observed—features that may impair their functional capacity within the hippocampal microenvironment. These morphological alterations could compromise key astrocytic functions, including: neurotransmitter clearance, regulation of ion homeostasis, formation and maintenance of the BBB, and support for neuronal plasticity, potentially leading to the onset of SRS.

In the subsequent part of our study, we assessed neuronal viability and overall cell death in OHCs cultured under different medium compositions. L-glutamine serves as a key metabolic substrate that supports cellular energy production, while a B-27 supplement provides essential neurotrophic factors, antioxidants, and lipids that support neuronal survival. Analysis of neuronal protein Tuj1, alongside measurements of factors released by OHCs, revealed that supplementation of standard culture medium with L-glutamine and/or B-27 supplement—either individually or in combination—did not improve OHC viability. Moreover, these additives had no significant effect on cell integrity or NO release throughout the culture period. Based on these findings, the culture medium for OHCs was not supplemented with either L-glutamine or B-27. Instead, OHCs were maintained in standard medium or in standard medium enriched with MSC-derived secretome to further investigate their specific effects on hippocampal tissue.

### 3.2. The Role of Growth Factors of Mesenchymal Stem Cells Secretome in Epilepsy

Cell-based therapies capitalize on the unique properties of MSCs, particularly their capacity to dynamically respond to inflammatory signals in the tissue microenvironment. The composition and activity of growth factors within the MSC secretome are influenced by multiple factors, including donor-specific characteristics, culture conditions, and environmental stimuli [59]. In the context of epilepsy, differences in the secretome profile, viability, and regenerative potential of MSCs derived from various donors may lead to variability in therapeutic outcomes, emphasizing the need for donor selection criteria and standardization of culture conditions. This is particularly valuable in the treatment of neurological disorders, where the complex and heterogeneous nature of the disease environment demands tailored cellular responses. The ability of MSCs to exert cytoprotective effects, preserve neural progenitor cells, and modulate immune responses highlights their therapeutic potential. Both preclinical and clinical studies have explored the genetic modification of MSCs to overexpress selected growth factors in order to enhance therapeutic efficacy [60,61,62].

In the present study, consistent concentrations of selected neurotrophic factors across four human MSC donors indicated low inter-donor variability, which supports their translational potential. Additionally, MSCs preserved their typical morphology, confirming cellular stability in different culturing media. The concentrations of certain factors in MSC secretome-enriched culture, such as BDNF, were found to approximate their physiological levels in cerebrospinal fluid [63], while others—such as HGF—substantially exceeded endogenous concentrations reported in brain tissue [64]. Existing data suggest that effective concentrations of BDNF and HGF required to activate their respective receptors and induce biological effects in hippocampal tissue in vitro range from 10 to 100 ng/mL [65,66,67]. Also, MSCs promote neuronal and glial survival via paracrine mechanisms, with neurotrophic factor levels comparable to those observed in our experiment [68]. Most of the analytes identified in the MSC-derived secretome exhibit neurotrophic, proneurogenic, and immunomodulatory properties, providing a strong rationale for the use of MSCs as a therapeutic strategy in neurobiological disorders, including epilepsy. For example, HGF has been shown to inhibit both apoptotic and astrogliotic processes, enhance learning and cognitive performance, and reduce seizure susceptibility in epileptic brain tissue [69,70]. Extensive preclinical evidence supports the anti-seizure efficacy of GDNF in animal epilepsy models [71,72,73,74]. While direct evidence for the anti-seizure properties of ANGPT-1 or BMP-4 remains lacking, both factors have been consistently implicated in promoting hippocampal neurogenesis and supporting neuronal repair [75,76,77,78,79,80]. Some of the trophic factors present in the MSC secretome may directly influence the balance between excitatory and inhibitory neurotransmission. NRG1 β1 plays a critical role in neuronal differentiation and modulates GABAergic interneurons, enhancing their inhibitory function [81,82] and restoring the balance between excitatory and inhibitory neurotransmission in response to seizures [83]. Additionally, the NRG1 may also display immunomodulatory influence [84]. Among the trophic factors investigated, BDNF appears to play a particularly complex and dualistic role in epilepsy [85]. On the one hand, BDNF has demonstrated promising potential in alleviating epileptic seizures [86,87,88,89]. On the other hand, studies have shown that BDNF may exacerbate epileptogenesis by heightening seizure susceptibility [90,91]. Elevated BDNF mRNA and protein levels observed in resected hippocampal tissue from TLE patients [92,93,94] may reflect a compensatory mechanism to reduce the excitability of hippocampal glutamatergic neurons [95]. Similarly, bFGF is reported to exert opposite effects on epileptic seizures [96]. This duality underscores the need for further research to elucidate the precise mechanisms by which trophic factors such as BDNF and bFGF influence epileptogenesis and seizure progression.

### 3.3. The Potential of the Mesenchymal Stem Cell Secretome to Preserve Neural Progenitor Populations and Its Immunomodulatory Properties in Organotypic Hippocampal Cultures from Temporal Lobe Epilepsy Mouse Model

Quantitative analysis of LDH activity in this study demonstrated a robust cytoprotective effect of MSC-CM across all experimental conditions. The sustained reduction in LDH release over time, observed in both epileptic and healthy tissue, underscores the capacity of the secretome to mitigate cellular damage irrespective of baseline tissue state, suggesting a broader tissue-stabilizing action. In epileptic slices, this effect may reflect attenuation of seizure-induced cytotoxic stress and stabilization of hippocampal integrity [97]. In control cultures, where no overt pathology is present, reduced LDH release results from enhanced cellular resilience and support of membrane integrity under baseline in vitro stress conditions [98]. In parallel, NO measurements performed in this study revealed a divergent, state-dependent immunomodulatory response: MSC-CM reduced NO levels in epileptic slices, suggesting a potential anti-inflammatory response, while promoting NO release in control cultures. The opposing NO secretion trends observed in healthy versus epileptic tissue reflect the differential baseline activation states of glial and immune-related pathways. In epileptic slices, where inflammation is already elevated [99,100], MSC-CM attenuates excessive NO production, whereas in non-pathological tissue, it may enhance physiological NO signaling linked to cellular maintenance and metabolic support [101]. This bidirectional effect implies that the secretome adapts its modulatory profile to the underlying condition of the tissue. This context-specific modulation is consistent with the adaptive and regulatory properties of MSC-derived factors, which can either suppress excessive inflammation [102] or support homeostatic signaling [103], depending on the baseline condition of the target tissue. Collectively, these findings confirm that MSC-CM exerts both cytoprotective and context-specific immunomodulatory effects in hippocampal slice cultures.

In the present study, culturing hippocampal slices for four and seven days in MSC secretome-enriched medium significantly attenuated the decrease in nestin levels compared to slices maintained in standard OHC medium. Nestin is a type IV intermediate filament protein and a marker specific to early-stage neural progenitors, which, upon further differentiation, can give rise to both neurons and glial cells [104,105]. Therefore, the reduced decline in nestin protein levels under the influence of the MSC-derived secretome observed in the present study can suggest an increased survival of nestin-positive progenitors; however, it does not constitute direct evidence for the promotion of hippocampal neurogenesis. However, the results of this study also demonstrate that medium conditioned by MSCs can affect the protein level of DCX—a microtubule-associated phosphoprotein characteristic of immature neurons and essential for their migration. Many reports indicate that epileptic seizures can severely affect the number of DCX-positive cells, but the direction of these changes is strongly stage-dependent [106,107,108]. For example, increased hippocampal neurogenesis and aberrant neuronal migration to the dentate hilus and molecular layer were observed right after SE onset [109,110]. In contrast, the chronic phase of epilepsy is often characterized by a reduction in DCX-positive cells in DG, which is associated with impairments in memory and cognitive function [107]. In the present study, the level of DCX remained unchanged in OHCs maintained in MSC secretome-enriched medium on the seventh day of culture when compared to its initial phase, and moreover, in the TLE group, it was increased compared to OHCs maintained in standard medium. Our results, therefore, suggest that MSC secretome-enriched culture medium may support the survival of DCX-positive cells. The observed increase in nestin and DCX levels did not correlate with the amounts of the neuronal marker Tuj1 in OHC homogenates, since its levels in all groups were shown to gradually decrease over seven days of OHC culture. Moreover, its concentrations in individual samples within every experimental group were characterized with great variability. Nevertheless, this study demonstrated that the MSC secretome may enhance the survival of nestin- and DCX-positive neural progenitors, with an indication of the neuronal fate of new cells. The MSC secretome may, therefore, support neuronal differentiation and help maintain neuronal function in epileptic hippocampal tissue.

Increased IL-6 levels are a recognized biomarker of various CNS disorders, including conditions associated with neuronal injury, such as stroke or traumatic brain injury [111], and are also commonly observed in patients with TLE [112]. The present study demonstrated a potential modulatory effect of MSC-derived secretome on IL-6, attenuating the TLE-induced elevation of its secretion from OHCs during the early phase of culture. The result obtained in our work may, therefore, indicate the anti-inflammatory effect of the secretome on isolated brain tissue affected by epileptogenesis. IL-6 exerts pleiotropic effects in the brain, and depending on the duration of its activity, concentration, and interactions with other pro-inflammatory mediators, it may either facilitate or suppress epileptic seizures [111]. Excessive activation of IL-6 signaling has been linked to exacerbation of neuroinflammation, maladaptive structural remodeling, and increased risk of drug-resistant seizures [113]. Conversely, IL-6 also exhibits neurotrophic properties, contributing to both developmental and adult neurogenesis, as well as the differentiation of neural and glial cells [114]. In the mature brain, IL-6 further supports neuronal microtubule stability and promotes axonal growth and regeneration [115]. In our study, IL-6 secretion decreased significantly over the following days of the experiment in hippocampal slices from all groups. However, its secretion remained at the highest level on day 7 in OHCs maintained in conditioned medium, and its concentration on that day was significantly higher than in TLE cultured in standard medium. Due to the overall low IL-6 concentrations after 7 days, however, the observed effect is of negligible biological significance, and increased IL-6 secretion may rather occur as a result of increased cell survival under the influence of the MSC secretome rather than being an indicator of neuroregenerative processes in isolated brain tissue.

In addition to a range of growth factors with well-established neurotrophic properties, MSCs secrete numerous anti-inflammatory mediators. In the present study, high levels of IL-4 were detected in MSC-conditioned culture medium. IL-4 is a pleiotropic cytokine with numerous suppressive effects on the inflammatory process [116] and maintaining proper neuronal activity and survival [117]. This effect is exerted mainly by modulating the function of glial cells, as IL-4 is known to have a profound effect on microglia [118] and astrocytes [119]. In the latent phase of epileptogenesis, this cytokine was shown to decrease the inflammatory response in reactive astrocytes by inhibiting NF-κB release [120].

In our study, the MSC secretome decreased the level of active p50 isoform of NF-κB and its precursor form (p105) at first and fourth day of hippocampal slice culture, and also the level of the precursor remained downregulated until the end of the experiment. Activation of NF-κB signaling in the brain is a common inflammatory biomarker of TLE, observed in both animal models and epileptic patients, and is associated with reactive gliosis and neurodegeneration [121]. NF-κB regulates the transcription of genes encoding pro-inflammatory cytokines such as IL-1β, IL-6, and TNF-α, and promotes their release from astrocytes and microglia, thereby contributing to further neuronal damage and the development of epileptic foci [122]. Interestingly, no significant changes in NF-κB p50 and its precursor (p105) levels were observed between the control and TLE group at the initial phase of OHC. This phenomenon may be directly related to a specific phase of epileptogenesis examined in the present study. It has been shown that, despite profound microglial activation during the latent phase of epilepsy, 14 days after induction of SE using pilocarpine, many inflammatory markers remained unaltered in experimental animals [4], and therefore, it is possible that activation of these specific pro-inflammatory components does not occur within the time frame chosen in our study. Decrease in NF-κB expression, observed during OHC course in the present study in epileptic hippocampal tissue, may be associated with the cumulative action of IL-4 and other anti-inflammatory cytokines, as well as the influence of some trophic factors. For example, it was shown that ANGPT-1 can cause inhibition of NF-κB expression by binding to TIE2 receptor, and activation of the Pi3K/Akt pathway [123]. Therefore, ANGPT-1 acts anti-inflammatorily and evokes survival, proliferation, and differentiation of cells (encourages neuroprotection and neurogenesis). Given that ANGPT-1 was present in the MSC secretome at high concentrations, exceeding 3000 pg/mL, which have been shown to be sufficient for the TIE2 receptor activation [124], it is therefore possible that ANGPT-1 contributed to the observed downregulation of NF-κB expression in the present study.

Given that astrocytes investigated in our model exhibit senescent characteristics, targeted interventions aimed at reversing or mitigating astrocytic dysfunction may enhance their neuroprotective roles and reduce tissue inflammation. A critical area for exploration is whether MSC-derived factors specifically modulate astrocytic senescence and how this modulation impacts the transition to the chronic epileptic state. Furthermore, the lack of significant changes in PV-positive and NPY-positive neuronal populations during the latent phase, in our experimental protocol, suggests that network hyperexcitability may stem from non-neuronal mechanisms, warranting a deeper investigation into glial-neuronal interactions and synaptic remodeling during epileptogenesis.

This study is the first to investigate the effects of the human MSC-derived secretome on epileptic hippocampal tissue specifically during the latent phase of epileptogenesis in an ex vivo model. This approach provides insight into the mechanisms through which epileptogenesis may potentially be halted or modulated before the onset of chronic seizures. Examining the cytoprotective and context-dependent immunomodulatory potential of the MSC secretome on epileptic tissue offers critical insights into phase-specific therapeutic strategies utilizing MSCs, particularly in determining whether there exists an epileptic stage-dependent window for therapeutic interventions involving MSCs that could disrupt the transition to chronic epilepsy, delay the onset of seizures, and potentially prevent their occurrence altogether. Taken together, our findings demonstrate that MSC-derived secretome exerts cytoprotective and context-dependent immunomodulatory effects in OHCs during the latent phase of epileptogenesis. By attenuating inflammation, reducing cellular stress, and supporting the preservation of neural progenitor markers, MSC-CM shows promise as a therapeutic strategy to modulate early pathological processes underlying epileptogenesis. These results support further investigation of MSC-CM as a disease-modifying intervention in epilepsy.

### 3.4. Limitations and Future Directions

While MSC therapies hold significant promise due to their anti-inflammatory, neuroprotective, and regenerative properties, several limitations must be addressed to optimize their clinical application. A major challenge lies in the heterogeneity of MSC populations, which can lead to variability in therapeutic efficacy. Clinical translation requires rigorous testing of the safety and reproducibility of MSC applications, with a focus on standardized dosing, and patient-specific factors such as age, sex, and epilepsy etiology [125]. Additionally, the delivery methods for MSCs or their secretome present logistical and biological challenges, including potential issues with targeted delivery, retention, and bioavailability within the affected tissue.

There are also certain methodological limitations within our experimental setup that should be acknowledged. The experimental readouts used in this study reflect selected aspects of complex biological processes. While they offer valuable insight into the cytoprotective and context-dependent immunomodulatory effects of the MSC secretome, they do not fully capture the multifactorial nature of tissue responses to injury and treatment. Furthermore, although we performed targeted characterization of the MSC-derived secretome using Luminex-based multiplex assays, deeper molecular profiling (e.g., mass spectrometry or extracellular vesicles cargo analysis) would be necessary to fully elucidate the range of bioactive factors and their potential mechanistic roles. Future studies should aim to integrate these expanded molecular insights to more precisely define the pathways by which MSC-CM exerts its context-specific modulatory effects.

Although the pilocarpine model of TLE is widely used, its implementation in NOD-SCID mice is limited, with only sparse literature available [126] and poses specific challenges due to the strain’s high seizure sensitivity and vulnerability to systemic stressors. In our setting, standard prolonged SE led to excessive mortality. Therefore, to ensure experimental feasibility, the protocol was adjusted by pharmacologically terminating SE shortly after onset. This approach deviates from classical pilocarpine protocols, in which SE is typically allowed to progress [127] to induce robust hippocampal pathology. This modification, while potentially limiting the extent of classical hippocampal pathology in the latent phase, allowed for reproducible induction of epileptogenesis, as evidenced by the emergence of SRS during the chronic phase under this protocol (data in preparation). Nevertheless, the results presented should be interpreted within a specific, defined experimental framework.

In our EEG setup, cerebellar referencing was chosen as a practical solution for capturing cortical seizure activity in vivo. Although the cerebellum is not an electrically neutral site, it is characterized by high-frequency neuronal firing and neurophysiological independence from forebrain structures [128], which minimizes its interference with cortical epileptiform discharges typically recorded in rodent models. This rationale, supported by prior studies utilizing cerebellar referencing [129], informed our electrode placement. In the pilocarpine model, cortical EEG discharges typically reflect underlying hippocampal epileptiform activity [130], whereas hippocampal seizures without cortical propagation may remain undetected using surface recordings [131]. Therefore, our configuration—though limited in localization precision—was sufficient for detecting seizure-related activity and correlating it with behavioral manifestations, which was the primary aim of EEG validation in this study.

The ex vivo nature of the employed OHC model, while allowing precise control over experimental conditions and direct assessment of tissue-specific responses to MSC-derived secretome, inherently limits the ability to fully recapitulate the complex systemic interactions present in vivo, such as immune cell infiltration, vascular responses, and long-range neuronal connectivity. In addition, the absence of dynamic physiological factors like circulating hormones, blood-brain barrier integrity, and multi-structural crosstalk may influence the cellular responses observed in OHCs. While OHCs retain much of the hippocampal cytoarchitecture, including synaptic circuitry and glial populations, their behavior outside the systemic environment may not entirely mirror in vivo pathophysiology, which should be considered when interpreting translational relevance. The dynamic and phase-specific nature of conditions like epilepsy necessitates precise timing of interventions, which remains difficult to determine. Further investigations that systematically address the aforementioned limitations can successfully pave the way in the future for developing tailored MSC-based therapies as a novel approach to managing epilepsy.

## 4. Materials and Methods

### 4.1. Animals

All experimental procedures were performed with the approval of the 1st Local Ethical Committee in Cracow, Poland (no. 415/2020 and 640/2022) in accordance with the 3R policy (Replacement, Reduction, Refinement) to minimize animal suffering and to reduce the total number of animals used.

### 4.2. Housing Conditions

Male and female NOD SCID mice (NOD.CB17-Prkdcscid/J) were originally purchased from Charles River (Sulzfeld, Germany) and bred in the animal housing facility of the Clinical Immunology and Transplantation Chair of Jagiellonian University Medical College. Both genders were used in equal proportion across all groups. Data from both sexes were pooled for analysis, as no sex-specific differences were observed in our study, and the aim was to maintain biological relevance and inclusivity. The mice were maintained in cages (four animals per cage) with high sanitary grade and micro-isolators, fed with heat-sterilized food and water, available ad libitum at a constant temperature of 22 °C, humidity of 55%, and light/dark cycle of 12/12 h. Eight-week-old mice with a minimum body weight of 20 g were included in the study.

### 4.3. Implantations of Transmitters

Seven days before TLE model induction, mice were anesthetized by i.p. injection of ketamine (75 mg/kg) (VetAgro, Poland) and xylazine (12.5 mg/kg) (Biowet Puławy, Poland). Anesthetized animals were placed in a stereotaxic frame (NeuroStar, RWD Life Science, Mainz, Germany) with a heating pad (32 °C) to maintain optimal body temperature. Before surgery, the shaved skin was disinfected using an iodine solution (#9322508, Braunol, Melsungen, Germany). Ophthalmic ointment (#37327, Puralube Ophtalamic Ointment, Dechra, Germany) was applied to the eyes throughout the procedure to prevent corneal drying. The animal’s head was placed horizontally, and a longitudinal incision along the midline axis of the skull was made with a scalpel blade to expose the bregma and lambda reference points. One transmitter was implanted subcutaneously on the upper back, while two electrodes were placed epidurally as follows: reference electrode AP −6.24 mm, ML 0 mm (above the cerebellum) and differential electrode AP −2.30 mm, ML −3.18 mm relative to Bregma (parietal-temporal lobe) [31,132,133,134]. A topical antiseptic (#34782, Polisept Vet, JM Sante Pharma, Będzin, Poland) was applied to prevent wound superinfection. Mice were allowed a seven-day postoperative recovery period before EEG signal acquisition to ensure adequate healing and to minimize potential confounding EEG effects of surgical stress. Following transmitter implantation, mice were housed separately to prevent mutual interference or injury due to the presence of subcutaneous devices. Animals were kept in individually ventilated cages (IVC, NexGen 500, Allentown, PA, USA) under standard laboratory conditions. Environmental enrichment (reusable shelters, nesting material, aspen-wood bedding, cardboard tunnels) was provided. Food and water were available ad libitum. Cage bedding and enrichment were refreshed weekly. For ten days before TLE model induction, mice were fed a mixture of autoclaved bread and milk powder mixed with sterile drinking water, which led to an increase in body weight, improved overall condition, and reduced mortality following drug administration.

### 4.4. Induction of Seizures in Mouse Model of Pilocarpine-Induced TLE

Thirty minutes before the administration of pilocarpine, all mice from the TLE model group received a single i.p. injection with 1 mg/kg of scopolamine butylbromide (#1610001, USP, Rockville, MD, USA) to block the peripheral effects of pilocarpine. Control group mice did not receive any drug treatment. Pilocarpine hydrochloride (#PHR1493-500, Merck Millipore, Darmstadt, Germany) was administered at a single dose of 295 mg/kg i.p. Immediately after pilocarpine administration, each mouse was placed in a sterile plastic beaker with a layer of bedding and put inside an incubator at 31 °C to avoid animal discomfort due to hypothermia. Seizure severity during pilocarpine-induced SE was assessed behaviorally using the modified Racine scale, which served as a primary inclusion criterion and outcome measure for effective model induction. The mice were closely monitored for manifestation of first epileptic changes as limbic motor symptoms corresponding to stage three or higher on the modified Racine scale (1972) [135], i.e., repeated rearing with forelimb clonus and loss of posture, generalized tonic-clonic seizures, wild running, and jumping. Immediately after the first onset of seizure, mice were given an i.p. injection of 5 mg/kg of diazepam (#350496, Hoffmann-LaRoche, Basel, Switzerland) to prevent further seizures. Mice were then placed back into their home cages and monitored daily for their behavior and overall welfare. Mice that did not exhibit limbic motor symptoms corresponding to stage three or higher on the modified Racine scale within 30 min post-pilocarpine administration were excluded from further experiments.

### 4.5. Radiotelemetry EEG Recording

EEG recordings served as an objective outcome measure for detecting epileptiform activity, characterized by sustained high-frequency, high-amplitude discharges. These data were used to validate seizure severity assessed behaviorally via the modified Racine scale. The EEG signal was acquired using a wireless Stellar Telemetry system (TSE Systems, Berlin, Germany). Recordings were made using BIOPAC AcqKnowledge 5.0 software (Biopac Systems Inc., Goleta, CA, USA) at a sampling rate of 200 Hz. EEG data were recorded at 20 and 30 min following intraperitoneal i.p. administration of pilocarpine to confirm the presence of epileptic activity. EEG epileptic discharges were identified by a burst of spiking with high-frequency and high-voltage synchronized profiles with a duration greater than ten seconds and amplitudes exceeding two times the background amplitude [31,126,136].

### 4.6. Immunofluorescence Using CLARITY

The Clear Lipid-exchanged Acrylamide-hybridized Rigid Imaging/Immunostaining/In situ hybridization-compatible Tissue-hYdrogel (CLARITY) method was performed using the MACS^®^ Clearing Kit (#130-126-719, Miltenyi Biotec, Bergisch Gladbach, Germany) according to the manufacturer’s instructions with slight modifications. This method enabled quantitative assessment of cell-type-specific structural changes in the hippocampus. The density of PV^+^/Tuj1^+^/DAPI^+^ interneurons in the CA1 region and GFAP^+^/DAPI^+^ astrocytes across CA1, CA3, and hilus subregions were evaluated as cellular outcome measures reflecting alterations in inhibitory circuitry and astroglial reactivity, respectively. Ten days after TLE model induction, mice from both the control and TLE model groups were decapitated and their brains were quickly removed and placed in an ice-cold working buffer (composed of 96% HBSS (#14025092, Thermo Scientific, Waltham, MA, USA); 3.5% glucose (#459560117, POCH, Gliwice, Poland); 0.5% penicillin and streptomycin (#15140-122, Thermo Scientific, MA, USA); HEPES (1M) (#H4034-100G, Sigma-Aldrich, Saint Louis, MO, USA) pH = 7.5). The hippocampi were carefully dissected from the brains using standard sterile surgical instruments. Subsequently, coronal hippocampal slices were prepared at a thickness of 300 μm using a McIlwain tissue chopper (TedPella, Redding, CA, USA) under ice-cold physiological conditions. Then slices were fixed at 4 °C in 4% PFA buffer (#P6148-500G, Sigma-Aldrich, MO, USA) for one hour. Residual PFA was removed by washing the slices three times in PBS (#14190250, Thermo Scientific, MA, USA) before tissue permeabilization in Permeabilization Solution (#130-126-719, Miltenyi Biotec, Bergisch Gladbach, Germany) for 24 h at room temperature (RT) with slow continuous rotation on a MACSmix™ Tube Rotator (#130-090-753, Miltenyi Biotec, Bergisch Gladbach, Germany). Two immunostainings were performed: with a primary antibody against GFAP (rat monoclonal IgG_2a_κ, #13-300, ThermoFisher, USA; 1:500) and the second with primary antibodies against PV (rabbit monoclonal IgG, #80561, Cell Signaling, Danvers, MA, USA; 1:500) and Tuj1 (mouse monoclonal IgG_1_, #MAB1637, Millipore, Darmstadt, Germany; 1:50). GFAP is a marker of astrocytes, used to assess glial reactivity [137], PV is a marker of fast-spiking GABAergic interneurons involved in inhibitory signaling [138], Tuj1 is a cytoskeletal protein marker of postmitotic neurons, indicative of neuronal differentiation [46]. Incubations with primary antibodies were performed for three days at 37 °C with horizontal shaking at 100 rpm. After incubation, unbound antibodies were removed by washing three times over a 24 h period at RT, with slow continuous rotation MACSmix™ Tube Rotator in Antibody Staining Buffer (#130-126-719, Miltenyi Biotec, Bergisch Gladbach, Germany), with buffer replaced every two hours. Tissue incubation with secondary antibodies was conducted under the same conditions over one day. AlexaFluor 488-conjugated goat anti-rat polyclonal IgG (#A-11006, Invitrogen, Waltham, MA, USA; 1:200) in GFAP-positive cells immunostaining and AlexaFluor 555-conjugated goat anti-rabbit polyclonal IgG (#A21428, Invitrogen, USA; 1:100) with AlexaFluor 488-conjugated goat anti-mouse polyclonal IgG (#A11001, Invitrogen, USA; 1:100) in PV- and Tuj1-positive cells immunostaining. The washing schedule for secondary antibodies was identical to the previous step. The stained tissues were dehydrated by incubation in a graded ethanol series: 50% (two hours), 70% (two hours), up to 100% (overnight) at RT with slow and continuous rotation. Following dehydration, tissue clearing was performed using MACS^®^ Clearing Solution (#130-126-719, Miltenyi Biotec, Bergisch Gladbach, Germany) for six hours at RT with slow and continuous rotation. Microscopic analysis was done after embedding the hippocampal slices in MACS^®^ Imaging Solution (#130-126-335, Miltenyi Biotec, Bergisch Gladbach, Germany). Sections were analyzed and photographed using a fluorescent microscope (Olympus BX53, Tokyo, Japan). To assess the profile of PV-immunopositive neurons, fluorescence microscopy analysis was performed on the CA1 subregion of the hippocampus. Quantification was based on the identification of triple-positive cells, defined as neurons co-expressing PV and Tuj1, along with a nuclear marker. DAPI is a fluorescent stain binding to DNA, used for nuclear visualization and cell counting [139]. For each animal, one coronal section per hippocampus (two sections per mouse) was analyzed, and all triple-positive cells located within the CA1 region were counted. The average number of PV^+^/Tuj1^+^/DAPI^+^ cells was normalized to 1 mm^2^ for each individual mouse, and results for each experimental group were visualized using bar plots with individual data points to illustrate the distribution and variability of the data.

To assess the profile and morphological features of GFAP-immunopositive cells, defined in this study as astrocytes, fluorescence microscopy analysis was performed on coronal hippocampal sections. Quantification was based on the identification of double-positive cells co-expressing GFAP and the nuclear marker DAPI. For each animal, one section per hippocampus (two sections per mouse) was analyzed. Within each hippocampus, representative fluorescence images were captured from three anatomically defined subregions: CA1, CA3, and hilus, resulting in six representative fields per animal. Cell counts and morphometric analyses were performed using ImageJ 1.54g software (NIH, Bethesda, MD, USA) across all six fields for each mouse. Results for experimental groups were visualized using bar plots with individual data points to illustrate the distribution and variability of the data. Quantitative statistical analysis was performed using the Mann–Whitney U test. A *p*-value < 0.05 was considered statistically significant.

### 4.7. Immunohistochemistry

Quantification of NPY-immunoreactive neurons in the hippocampal hilus served as the primary outcome measure for assessing changes in inhibitory interneuron populations during the latent phase of epileptogenesis. Ten days post TLE model induction, animals were deeply anesthetized with a cocktail of ketamine (115 mg/kg) (VetAgro, Lublin, Poland) and xylazine (10 mg/mL) (Biowet Puławy, Puławy, Poland) solution i.p. and transcardially perfused with ice-cold 0.9% NaCl solution (#08646871, Braun, Frankfurt am Main, Germany), followed by 4% paraformaldehyde solution (#P6148-500G, Sigma-Aldrich, MO, USA) prepared in-house using 0.1 M phosphate buffer (pH 7.4). After perfusion, brains were removed, placed in the same fixative for 24 h at 4 °C, then transferred to 1M PBS solution (#14190144, Thermo Scientific, MA, USA) at 4 °C. Cryoprotection was achieved by immersion in a 20% sucrose solution for one day. Subsequently, the brains were frozen on dry ice, and 40 μm thick frontal free-floating sections were cut using a cryostat (CryoStar NX70 Cryostat, Thermo Scientific, USA). To minimize inter-assay variability, all experimental groups were processed simultaneously under the same conditions. After thorough washing in PBS, tissue sections were incubated with 1% hydrogen peroxide (diluted from a 30% stock solution; # 1072090250, Sigma-Aldrich, MO, USA) for ten minutes. Following another wash, the sections were incubated in a blocking solution containing 5% Normal Swine Serum (product of University Slaughterhouse), 1% Bovine Serum Albumin (BSA) (# A9647, Sigma-Aldrich, MO, USA), and 0.3% Triton X-100 (TX-100) (#X100, Sigma-Aldrich, MO, USA) for 20 min. Then, the slices were incubated overnight with primary rabbit antiserum against NPY (#8182, previously validated [140], 1:1000) diluted in 1% BSA and 0.3% TX-100. NPY is a neuropeptide expressed in inhibitory interneurons, involved in synaptic modulation and neuroprotection; its levels reflect adaptive or pathological plasticity in the hippocampus [141]. The next day, sections were washed and incubated for one hour with biotinylated secondary antibody (goat anti-rabbit polyclonal IgG, #BA-1000, Vector Laboratories, Newark, CA, USA; 1:1000) diluted in 1% BSA and 0.3% TX-100. Next, slices were washed and incubated with avidin-biotinylated peroxidase (#PK-4000, Vectastain ABC Peroxidase Standard Kit, Vector Laboratories, CA, USA) for one hour. Staining was then revealed by incubating the sections with 3,3′-diaminobenzidine (#D5637, Sigma-Aldrich, MO, USA) and hydrogen peroxide. Finally, sections were mounted on gelatin-coated slides and cover-slipped. For each animal, one coronal brain section was analyzed, and all NPY-immunoreactive cells located within both hilus regions of the dorsal hippocampal formation were counted. Quantification was performed by investigators blinded to the experimental groups. The total number of NPY-positive cells per animal was normalized to 1 mm^2^. Results for each experimental group were visualized using bar plots with individual data points to illustrate the distribution and variability of the data. Quantitative statistical analysis was performed using the Mann–Whitney U test. A *p*-value < 0.05 was considered statistically significant.

### 4.8. Establishment of OHCs

In the TLE, the latent phase of epileptogenesis is defined as the seizure-free interval following SE and preceding the onset of SRS. Based on prior studies, this phase is characterized by progressive molecular and structural changes, including neuroinflammation, interneuron loss, glial activation, and early synaptic reorganization, without overt seizure activity [127,142,143]. The two-week post-SE timeframe has been widely used in the literature to elucidate these events [24,144,145]. In this study, the 10-day post-SE timepoint was selected as a representative stage of the latent phase for evaluating cellular and molecular correlates of epileptogenesis.

The OHCs were established from the hippocampus of adult, male and female NOD SCID mice aged nine weeks, ten days after TLE model induction, according to the protocol described by Gogolla et al. (2006) [146] with slight modifications. Briefly, after rapid cervical dislocation and decapitation, isolated brains were immediately placed in ice-cold working buffer containing 96% HBSS (#14025092, Thermo Scientific, MA, USA); 3.5% glucose (#459560117, POCH Gliwice, Poland); 0.5% penicillin and streptomycin (#15140-122, Thermo Scientific, MA, USA) and HEPES (1M) (#H4034-100G, Sigma-Aldrich, MO, USA) pH = 7.5). Hippocampi were carefully dissected using standard sterile surgical instruments. Subsequently, coronal hippocampal slices (300 μm thick) were prepared using a McIlwain tissue chopper (TedPella, Redding, CA, USA) under ice-cold physiological conditions. The hippocampal sections were rinsed with ice-cold working buffer, transferred onto ThinCert™ membranes (#657641, Greiner Bio-One, Kremsmünster, Austria), and placed in 6-well plates (#657185, Greiner Bio-One, Kremsmünster, Austria) filled with 1 mL of standard OHC medium composed of 25% MEM 2x (#11935-046, Thermo Scientific, MA, USA); 25% HBSS (#14025092, Thermo Scientific, MA, USA); 25% horse serum (#26050-088, Thermo Scientific, MA, USA); 22% sterilized, 18.2 MΩ re-distilled water; 0.5% penicillin and streptomycin (#15140-122, Thermo Scientific, MA, USA) and 2.5% of 10 mM TRIS (#TRS001.1, BioShop, Burlington, ON, Canada)/25mM HEPES (#H4034-100G, Sigma-Aldrich, MO, USA) solution to maintain the constant pH.

In initial experiments, aimed establishing optimal formulation of the OHC medium, standard composition of medium was additionally supplemented with L-glutamine (at final concentration of 2 mM; #25030-081, Thermo Scientific, MA, USA) and B-27 supplement without vitamin A (0.5× concentrated; #12587010, Thermo Scientific, MA, USA), added to the standard medium separately or jointly. Four hippocampal slices were cultured in each well for 14 days (in initial experiments) or 7 days (in the experiment with MSC secretome investigation) at 37 °C in an incubator with 5% CO_2_. The media were exchanged according to the following schedule: on days 1, 2, and 5 for 7-day cultures, or on days 1, 3, 5, 7, 10, and 12 for 14-day experiments. The 14-day cultures were used to optimize medium composition under prolonged in vitro conditions, which allowed assessment of long-term tissue stability. Following the medium optimization process, a 7-day culture duration was selected for subsequent experiments assessing the effects of the MSC-CM, as it ensured optimal tissue viability and consistency of experimental conditions.

### 4.9. Preparation of MSC-CM for OHC Experiments

Human MSC lines derived from Wharton’s jelly of the umbilical cord from four different donors, used at passage five, were obtained from the Tissue and Cell Bank Laboratory, Department of Transplantation, Chair of Clinical Immunology and Transplantation, Jagiellonian University Medical College. All procedures involving human cells were approved by the Bioethics Committee of the Jagiellonian University (no. 1072.6120.95.2021). MSCs were cultured in flasks precoated with 0.1% gelatin in standard MSC growth medium, containing DMEM low glucose (1g/L) (#L0066-500, Biowest, Nuaillé, France) supplemented with 7.5% human platelet lysate (#BC0190020, Macropharma, Wrocław, Poland), 2 mM L-glutamine (#25030-081, Thermo Scientific, MA, USA), 2 IU/mL heparin (Polfa Warszawa, Warsaw, Poland), and 50 μg/mL gentamicin sulfate (#BE02-012E, Lonza, Basel, Switzerland). Cell culture was conducted at 37 °C in an atmosphere containing 5% CO_2_ and 95% humidity.

To study the effects of the MSC secretome on OHCs derived from mice in the TLE model, 6 × 10^3^/cm^2^ of MSCs from a single preselected donor were seeded onto gelatin pre-coated 175 cm^2^ surface culture flasks. At approximately 80–85% confluence, the standard culture medium was replaced with 10 mL of OHC medium. After 48 h, MSC-CM was collected, centrifuged (1000 rpm for 5 min at 4 °C), and frozen at −20 °C. The MSC culture was maintained for an additional 48 h using 10 mL of fresh OHC medium. After this time, MSC-CM was preserved for further analysis as described above. One milliliter of thawed and pre-heated MSC-CM from the second conditioning phase was added to the wells containing hippocampal slices on six well plates and was exchanged according to the same scheme as standard OHC medium, at days 1, 2, 5, and 7 of culture. At these timepoints, samples of standard and MSC-CM were collected, briefly centrifuged (1000 rpm, two min), and frozen at −20 °C for biochemical assays. The 48 h collection period was selected to maximize the concentration of bioactive factors in the MSC-conditioned medium without compromising cell viability. Since the MSC were not genetically modified to enhance trophic factor secretion and the medium was not further concentrated or purified, extended culture in OHC medium at high confluence was necessary to ensure adequate secretome enrichment. The 48 h window represented the optimal balance, providing a high secretory activity while avoiding cell detachment and maintaining monolayer integrity.

### 4.10. Evaluation of LDH Activity and NO Level in the Culture Media of OHCs

LDH was assessed as a cytosolic enzyme released upon loss of membrane integrity, serving as a marker of tissue injury [147], while NO was measured as a reactive signaling molecule; its elevated levels typically indicate inflammatory activation and oxidative stress [148]. However, nitric oxide also plays a key role in physiological cell signaling, including neurotransmission and vascular regulation, which should be taken into account when interpreting its dynamics. The concentrations of LDH and NO in the culture medium were used as quantitative outcome measures reflecting tissue damage and cellular stress.

The activity of LDH in culture media was measured with the Cytotoxicity Detection Kit (#4744926001, Roche Diagnostic, Mannheim, Germany). Culture media were collected according to the media changing schedule, on days 2, 5, 7, 10, and 14. Fifty microliters of each sample were dispensed into a 96-well plate, followed by the addition of an equal volume of a reagent mixture prepared according to the manufacturer’s protocol. After incubation at 37 °C, a red color developed, the intensity of which was directly proportional to the number of damaged or dead cells. The absorbance was measured at a wavelength of 490 nm with a reference wavelength of 600 nm using a Spark 10M spectrophotometer (TECAN, Männedorf, Switzerland).

The level of NO was detected using a colorimetric Griess test. Fifty microliters of culture medium were mixed with an equal volume of Griess reagent (0.1% N-1-naphthylethylenediamine dihydrochloride (#222488, Sigma-Aldrich, MO, USA) and 1% sulfanilamide (#S-9251, Sigma-Aldrich, MO, USA) in 5% phosphoric acid (#569150111, POCH, Poland)) in a 96-well plate. The intensity of the formed color was measured at a wavelength of 540 nm using a Spark 10M spectrophotometer (TECAN, Männedorf, Switzerland). Results were calculated as fold change vs. control (Ctrl/Std) at Day 2. Two-way ANOVA followed by Tukey’s post hoc test was used to statistically evaluate differences between experimental groups within each time point. Differences between the same groups between individual experimental days were evaluated using one-way ANOVA and Tukey’s post hoc test. A *p*-value < 0.05 was considered statistically significant. All results are presented as mean ± SEM.

### 4.11. LUMINEX^®^ Multiplex Assays

The Human Premixed Multi-Analyte Kit (#LXSAHM, Bio-Techne, Minneapolis, MN, USA) was used to measure the concentrations of selected bioactive molecules present in the MSC secretome, including ANGPT-1, BMP4, GDNF, IL-4, NRG1 β1, BDNF, bFGF, and HGF. These factors were selected based on their known neurotrophic, anti-apoptotic, immunomodulatory, pro-angiogenic, and regenerative properties, which are relevant to the pathophysiology of epilepsy and the potential therapeutic mechanisms [69,70,71,75]. Measuring their concentrations in culture medium aimed to characterize the therapeutic potential of MSC-derived secretome.

The Mouse Premixed Multi-Analyte Kit (#LXSAMSM, Bio-Techne, MN, USA) was used to determine the cytokine release profile in the OHC slice culture medium obtained from mice with pilocarpine-induced seizures and control animals. The slices were cultured in standard or MSC-CM. The concentrations of the following cytokines were measured: interferon-γ (IFN-γ), interleukin-2 (IL-2), interleukin-6 (IL-6), interleukin-12 p70 (IL-12 p70), tumor necrosis factor-α (TNF-α), interleukin-1β (IL-1β), interleukin-4 (IL-4), interleukin-10 (IL-10), and interleukin-13 (IL-13). Levels of secreted cytokines in the culture medium of OHCs were measured to evaluate the responses of hippocampal tissue to MSC-conditioned medium exposure.

In both cases, samples were diluted 1:1, aliquoted onto 96-well plates along with appropriate blanks and standards, and mixed with an antibody-coated magnetic bead mixture. Fluorescence signal was measured with the Luminex^®^ 100/200™ (Luminex Corporation, Northbrook, IL, USA). Results were expressed as pg/mL of medium. Statistical evaluation of the results was performed using two-way ANOVA with Tukey’s post hoc test. Differences between the same groups between individual experimental days were evaluated using one-way ANOVA and Tukey’s post hoc test. A *p*-value < 0.05 was considered statistically significant.

### 4.12. Western Blot Analysis

Western blot analyses quantified hippocampal protein levels of nestin, DCX, and Tuj1 as markers of neurogenesis and neuronal differentiation, and NF-κB subunits (p105/p50) as indicators of inflammatory signaling activation.

Wells and culture inserts, containing OHCs, were rinsed twice with PBS without calcium and magnesium prior to harvesting hippocampal slices. Next, the brain tissues were lysed in 2% sodium dodecyl sulfate (SDS) (#SDS002.500, BioShop, Canada) in dH_2_O and centrifuged at 13,000× *g* for 20 min at room temperature. Protein concentrations in the supernatants were determined using the bicinchoninic acid (BCA) method [149] with the Pierce BCA Protein Assay Kit (#23227, Thermo Scientific, MA, USA). Samples, containing equal amounts of protein, were mixed in a 4:1 ratio (*v*/*v*) with NuPAGE LDS Sample buffer (#NP0007, Invitrogen, USA) with the addition of 10% Bond Breaker TCEP solution (#77720, Thermo Scientific, MA, USA) and incubated for ten minutes at 70 °C.

Proteins were separated in 12% polyacrylamide gels under a constant voltage of 120 V, transferred (at 90 V for one hour) onto PVDF membranes (#1620177, Bio-Rad, Hercules, CA, USA), blocked in 1% BSA in TTBS (TRIS-buffered saline with 0.05% Tween 20) for one hour and incubated overnight at 4 °C with primary antibodies raised against DCX (rabbit polyclonal IgG, #48-1200, Thermo Fisher Scientific, 3 μg/mL), nestin (mouse monoclonal IgG_1_κ, #MA1-110, Thermo Fisher Scientific, 1:1000), NF-κB p105/p50 (rabbit recombinant monoclonal, #ab32360, Abcam, London, UK, 1:1000), Tuj1 (mouse monoclonal IgG1, #MAB1637, Millipore, Germany; 1:1000). On the following day, membranes were washed three times in TTBS for 15 min and incubated for one hour with appropriate HRP-conjugated secondary antibodies at a dilution of 1:3000 (mouse IgG kappa binding protein, #sc-516102; mouse anti-rabbit, #sc-2357 Santa Cruz Biotechnology, Dallas, TX, USA) at RT. After three further washings in TTBS, the membranes were developed with SuperSignal West Pico Chemiluminescence Substrate (#34580, Thermo Fisher Scientific, USA) in ChemiDoc MP Imaging System (Bio-Rad, USA). The chemiluminescence signal was analyzed using Image Lab 6.1 software (Bio-Rad, USA). Beta-Actin served as the internal loading control and was detected with HRP-conjugated mouse monoclonal IgG_1_ (#sc-47778, Santa Cruz Biotechnology, USA; 1:1000). Results were calculated and presented as fold change vs. control group (Ctrl/Std) at first day (Day 1) of OHC. For statistical evaluation, two-way analysis of variance and Tukey’s post hoc test were implemented to compare results between groups on the same experimental day. One-way ANOVA and Tukey’s post hoc test were used to compare results from the same experimental group on different days of OHC culture. A *p*-value < 0.05 was considered statistically significant. All results are presented as mean ± SEM.

A total of approximately 128 mice were used in this study. Due to the multistage experimental design, individual analyses were conducted on separate subgroups of animals, resulting in in vivo group sizes ranging from 5 to 9 mice per condition, depending on the analysis. Approximately six organotypic slices were prepared from each mouse hippocampus. Four slices, comprising equal contributions from male and female mice, were pooled per membrane to ensure sex balance within samples. For Western blot and luminometric analyses, sample sizes ranged from 6 to 10 cultures per group, depending on the molecular marker. For colorimetric assays, 13 to 15 OHCs were analyzed per condition and were allocated to experimental analysis accordingly. MSC-CM was collected from six independent cultures derived from the selected donor batch for use in all subsequent experiments. Detailed group sizes for each analysis are provided in the corresponding figure legends. Group sizes were determined based on standard power considerations for preclinical neuroscience studies and were selected to achieve an estimated statistical power of 80% at a significance level of α = 0.05, assuming moderate effect sizes and variability consistent with prior pilocarpine-induced TLE models. This approach ensured reliable detection of biologically meaningful differences while adhering to the 3R principle.

### 4.13. Statistical Analysis

All statistical analyses were performed using GraphPad Prism software 10.5.0 (GraphPad Software, San Diego, CA, USA) and Statistica 12 software (TIBCO Software Inc.). Prior to statistical comparison, outliers were detected using the robust nonlinear regression-based ROUT method (Q = 1%) or Grubbs’s test and excluded from further analysis. The normality of the data was assessed using the Shapiro–Wilk test, and the homogeneity of variances was confirmed using Levene’s test. For comparisons between two independent groups (Ctrl vs. TLE), the Mann–Whitney U test was used (Figure 2B, Figure 3B and Figure 4B–G). For experiments including two factors, two-way ANOVA followed by Tukey’s post hoc test was applied. Differences between the same group between individual experimental days were evaluated using one-way ANOVA and Tukey’s post hoc test (Figure 6, Figure 7 and Figure 8; Supplementary Figure S1B–D). Results are presented as mean ± SEM unless stated otherwise; for Table 1, values are reported as mean ± SD. Statistical significance was set at *p* < 0.05. Individual data points are shown in all bar graphs to illustrate variability within each group.

## Figures and Tables

**Table 1:** Comparison of secretion profiles between MSCs isolated from four different human donors. Secretion of selected neurotropic and anti-inflammatory factors by mesenchymal stem cells (MSCs) at fifth passage in standard medium for organotypic hippocampal cultures (OHCs) after 48 h was measured with multiplex immunoassay. Results are presented as mean concentrations ± SD, number of cell cultures *n* = 2.

MSC	Analyte Concentration [pg·mL^−1^] M ± SD							
	ANGPT-1	BDNF	bFGF	BMP-4	GDNF	HGF	IL-4	NRG1 β1
Donor 1	3580.2 ± 903.7	185.1 ± 53.9	14.7 ± 0.1	99.9 ± 3.0	31.1 ± 3.8	3118.0 ± 1306.7	357.9 ± 36.7	320.4 ± 29.0
Donor 2	1380.5 ± 228.1	199.3 ± 32.5	18.4 ± 4.5	83.1 ± 12.9	42.1 ± 10.4	2028.9 ± 775.0	264.3 ± 39.1	238.0 ± 36.1
Donor 3	1650.8 ± 454.2	290.8 ± 78.9	31.0 ± 12.3	98.3 ± 0.3	87.5 ± 6.7	3112.0 ± 1119.3	371.5 ± 35.8	350.9 ± 31.9
Donor 4	2403.9 ± 742.5	341.6 ± 128.6	40.0 ± 7.6	128.7 ± 0.0	75.9 ± 16.5	3247.8 ± 164.4	462.2 ± 105.1	393.3 ± 74.3
**Mean**	**2253.9 ± 582.1**	**254.2 ± 73.5**	**26.0 ± 6.1**	**102.5 ± 4.1**	**59.2 ± 9.4**	**2876.6± 841.3**	**364.0 ± 54.2**	**325.6 ± 42.8**

## Data Availability

The original contributions presented in this study are included in the article or Supplementary Materials. Further inquiries can be directed to the corresponding author.

## Supplementary Materials

The following supporting information can be downloaded at: https://www.mdpi.com/article/10.3390/ijms27010265/s1.

## Abbreviations

ANGPT-1	angiopoietin-1
BDNF	brain derived neurotrophic factor
bFGF	basic fibroblast growth factor
BMP-4	bone morphogenetic protein 4
DCX	doublecortin
GDNF	glial-derived neurotrophic factor
HGF	hepatocyte growth factor
IL-4	interleukin-4
IL-6	interleukin-6
LDH	lactate dehydrogenase
MSCs	Mesenchymal Stem Cells
MSC-CM	Mesenchymal Stem Cell-conditioned medium
NF-κB	nuclear factor kappa-light-chain-enhancer of activated B cells
NO	nitric oxide
NRG1 β1	neuregulin-1 β1
OHCs	Organotypic Hippocampal Cultures
TLE	Temporal Lobe Epilepsy
Tuj1	β-tubulin III
