# Supplementary material for: Cytoprotective and Immunomodulatory Properties of Mesenchymal Stem Cell Secretome and Its Effect on Organotypic Hippocampal Cultures in Mouse Model of Temporal Lobe Epilepsy
Source: Int J Mol Sci. 2025 Dec 26;27(1):265. doi: 10.3390/ijms27010265 (PMC12785294; doi:10.3390/ijms27010265)
Supplement: Supplementary file 1 [file ijms-27-00265-s001.zip › ijms-4019813-SI.pdf]

Supplementary Materials

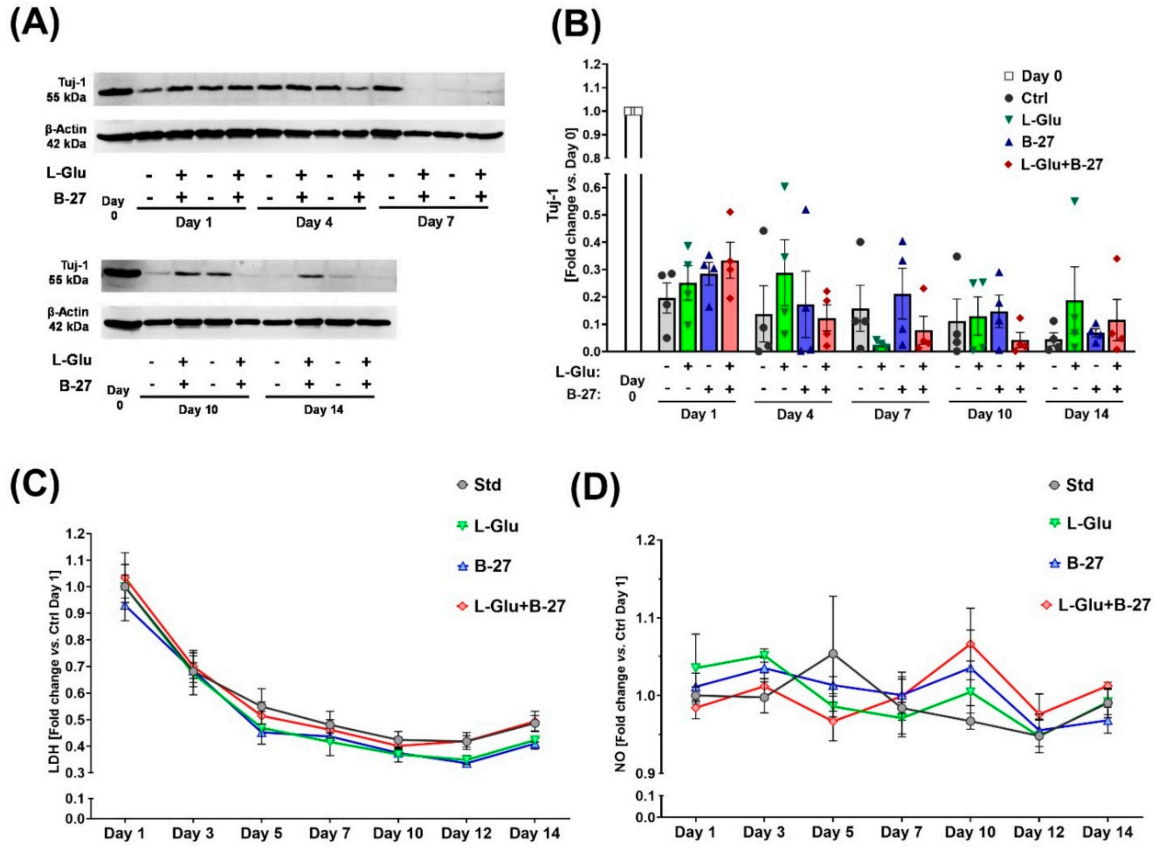

Supplementary Figure 1. Establishment of the culture medium formulation for OHCs. The culture medium was supplemented with L-glutamine, B-27 supplement, or their combination, and the cultures were maintained for 14 days. The viability of the cultured tissue was assessed based on the expression of the neuronal marker β-tubulin III (Tuj1), the level of lactate dehydrogenase (LDH) activity, and the concentration of nitric oxide (NO) in the culture medium. (A) and (B) Western blot analyses of Tuj1 in OHCs after 1, 4, 7, 10, 14 days of culture, along with representative sample membrane images. Results were presented as fold change *vs*. concentration of Tuj1 at day 0. Results presented as mean ± SEM, n=4, (C) LDH activity measured by tetrazolium salt test and (D) NO level measured by Griess test in the culture medium. Colorimetric tests were provided in time points resulting from the pattern of medium changes in the culture, this is: at 1, 3, 5, 7, 12 and 14 days of the culture. Statistical analysis was performed using two-way ANOVA test and Tukey's *post hoc* test. Results presented as mean ± SEM, n=4. Results are presented as fold change vs. control group from each day, cultured in standard OHCs medium.
